# Single-cell RNA sequencing unveils Lrg1's role in cerebral ischemia‒reperfusion injury by modulating various cells

**DOI:** 10.1186/s12974-023-02941-4

**Published:** 2023-11-30

**Authors:** Zhaohui Ruan, Guosheng Cao, Yisong Qian, Longsheng Fu, Jinfang Hu, Tiantian Xu, Yaoqi Wu, Yanni Lv

**Affiliations:** 1 Department of Pharmacy, The First Affiliated Hospital of Nanchang University, Nanchang, China; 2 College of Pharmacy, Hubei University of Chinese Medicine, Wuhan, China; 3 School of Clinical Medicine, Nanchang University, Nanchang, China

**Keywords:** Single-cell RNA-seq, *Lrg1* knockout, Cerebral ischemia–reperfusion injury, Microglial cell

## Abstract

**Background and purpose:**

Cerebral ischemia‒reperfusion injury causes significant harm to human health and is a major contributor to stroke-related deaths worldwide. Current treatments are limited, and new, more effective prevention and treatment strategies that target multiple cell components are urgently needed. Leucine-rich alpha-2 glycoprotein 1 (Lrg1) appears to be associated with the progression of cerebral ischemia‒reperfusion injury, but the exact mechanism of it is unknown.

**Methods:**

Wild-type (WT) and *Lrg1* knockout (*Lrg1*^−/−^) mice were used to investigate the role of Lrg1 after cerebral ischemia‒reperfusion injury. The effects of *Lrg1* knockout on brain infarct volume, blood‒brain barrier permeability, and neurological score (based on 2,3,5-triphenyl tetrazolium chloride, evans blue dye, hematoxylin, and eosin staining) were assessed. Single-cell RNA sequencing (scRNA-seq), immunofluorescence, and microvascular albumin leakage tests were utilized to investigate alterations in various cell components in brain tissue after *Lrg1* knockout.

**Results:**

Lrg1 expression was increased in various cell types of brain tissue after cerebral ischemia‒reperfusion injury. *Lrg1* knockout reduced cerebral edema and infarct size and improved neurological function after cerebral ischemia‒reperfusion injury. Single-cell RNA sequencing analysis of WT and *Lrg1*^−/−^ mouse brain tissues after cerebral ischemia‒reperfusion injury revealed that *Lrg1* knockout enhances blood‒brain barrier (BBB) by upregulating claudin 11, integrin β5, protocadherin 9, and annexin A2. *Lrg1* knockout also promoted an anti-inflammatory and tissue-repairing phenotype in microglia and macrophages while reducing neuron and oligodendrocyte cell death.

**Conclusions:**

Our results has shown that Lrg1 mediates numerous pathological processes involved in cerebral ischemia‒reperfusion injury by altering the functional states of various cell types, thereby rendering it a promising therapeutic target for cerebral ischemia‒reperfusion injury.

**Supplementary Information:**

The online version contains supplementary material available at 10.1186/s12974-023-02941-4.

## Highlights

1. Upregulated Lrg1 expression is observed in various cells in brain after cerebral ischemia‒reperfusion injury.
2. *Lrg1* knockout can attenuate brain damage and blood‒brain barrier dysfunction following cerebral ischemia‒reperfusion injury.
3. *Lrg1* knockout can alter the functional state of microglial cells towards the anti-inflammatory M2 phenotype.
4. *Lrg1* knockout can modulate the metabolic status of multiple cell types.

**Supplementary Information:**

The online version contains supplementary material available at 10.1186/s12974-023-02941-4.

## Introduction

Ischemic stroke is one of the most common diseases with high mortality and disability rates worldwide [1]. Ischemic stroke patients may suffer from cerebral ischemia‒reperfusion injury after the blood supply is restored, further exacerbating neuronal death and neurological dysfunction and severely impeding the recovery of acute ischemic stroke patients [2]. The mechanisms underlying cerebral ischemia‒reperfusion injury remain unclear, and agents that effectively treat or prevent cerebral ischemia‒reperfusion injury are not currently available [3]. Therefore, further exploration is needed to identify drugs potentially useful in the treatment of cerebral ischemia‒reperfusion injury.

Cerebral ischemia‒reperfusion injury is an extremely complex process that involves disruption of the blood‒brain barrier (BBB), brain inflammation, and increased neuronal apoptosis, which are associated with various cellular components in the brain. BBB breakdown after cerebral ischemia‒reperfusion injury is mainly manifested by a decrease in the connectivity between the cell components of the BBB (including pericytes, endothelial cells, astrocytes, and basement membrane) and an increase in leakage [4, 5]. The progression of cerebral ischemia‒reperfusion injury is also associated with various immune cells, which play a dual role in the progression of cerebral ischemia‒reperfusion injury. Immune cells, including microglia and macrophages, secrete tissue repair factors and engulf dead cells to facilitate brain tissue recovery after cerebral ischemia‒reperfusion injury [6]. On the other hand, various inflammatory factors, including IL-6 and TNF-α, exacerbate the progression of cerebral ischemia‒reperfusion injury [7, 8]. Because the disease process involves multiple cellular components, the identification of treatment targets for multiple cellular components may be a better approach for preventing and treating cerebral ischemia‒reperfusion injury [6].

Single-cell RNA sequencing (scRNA-seq), a cutting-edge research technique, provides new insights into complex diseases involving multiple cellular components, such as cerebral ischemia‒reperfusion injury. Researchers have utilized scRNA-seq to reveal the intricate mechanisms in brain tissue after cerebral ischemia‒reperfusion injury, including the finding that the polarization of microglia into M1 or M2 phenotypes may not be simply defined [9]. ScRNA-seq facilitates the comprehensive understanding of the mechanisms underlying cerebral ischemia‒reperfusion injury or the therapeutic efficacy of target interventions.

Lrg1 is considered a signaling molecule that is potentially induced after cerebral ischemia‒reperfusion injury. Researchers have shown that Lrg1 is associated with increased brain infarct volume, brain cell apoptosis, and enhanced autophagy, aggravating cerebral ischemia reperfusion injury [10, 11]. However, the precise role and underlying mechanisms of Lrg1 in the process of cerebral ischemia‒reperfusion injury remain elusive. There is accumulating evidence suggesting that Lrg1 may have an impact on various cellular components in brain tissues, including neuronal and endothelial cells. Specifically, Lrg1 was shown to induce activation of the TGF-β pathway in vascular endothelial cells and promote endothelial cell mitosis in a middle cerebral artery occlusion (MCAO) mouse model [12]. Additionally, recent literature has highlighted the potential role of Lrg1 as a mediator of the "niche", stimulating the growth of nearby connective tissue cells and providing a niche for lung metastasis [13]. However, the precise mechanisms underlying its effects on cerebral ischemia‒reperfusion injury remain unclear. Specifically, it remains unclear whether the effect of Lrg1 on cerebral ischemia‒reperfusion injury progression is related to various cellular components in brain tissue.

In this study, we employed *Lrg1* knockout mice to investigate the role of Lrg1 in cerebral ischemia‒reperfusion injury. Utilizing various techniques, such as scRNA-seq, immunofluorescence, and BBB permeability assays, we revealed continuous upregulation of Lrg1 expression across various cell components of the brain in the pathologic process of cerebral ischemia‒reperfusion injury. Our preliminary data showed that *Lrg1* knockout had little effect on cerebral vasculature of mice, and with no effect on the pathological changes of blood vessel and the normal physiological activities. Furthermore, a comparative analysis of cerebral ischemia‒reperfusion injury-induced brain infarct size, brain edema volume, and neurofunctional status between *Lrg1* knockout and wild-type mice demonstrated that *Lrg1* deletion confers neuroprotection against cerebral ischemia‒reperfusion injury-induced brain tissue damage. Our comprehensive elucidation of the role of Lrg1 in the progression of cerebral ischemia‒reperfusion injury based on scRNA-seq data indicates its ability to modulate of multiple cellular components within the brain, including perivascular cells, microglial cells, and endothelial cells, highlighting Lrg1 as a promising therapeutic target for the treatment of cerebral ischemia‒reperfusion injury.

## Methods

### Animals and ethics approval

Wild-type C57BL/6 mice (male, 8‒10 week) weighing 18‒22 g were obtained from the College of Veterinary Medicine Yangzhou University (Institute of Comparative Medicine) (certificate No. SCXK 2021-0003). Conventional *Lrg1* knockout mice (*Lrg1*^−/−^) on a C75BL/6 background were originally generated and mated at Cyagen Biosciences Corporation (strain number: C57BL/6 J, strain name: KOCMP‒76905-Lrg1-B6N-VA). The Institutional Animal Care and Use Committee of The First Affiliated Hospital of Nanchang University (Nanchang University, Nanchang, China) approved all procedures performed in this study (Ethics approval number: IACUC Issue No: CDYFY-IACUC-‒202306QR028).

### *Lrg1* knockout mice

*Lrg1* knockout mice were obtained from Cyagen Biosciences Corporation (strain number: C57BL/6N-Lrg1^em1cyagen^, strain name: KOCMP-76905-Lrg1-B6N‒VA). Exon 1 and the TGA stop codon in exon 2 (Transcript: NSMUST00000041357) were selected as target sites, and ~ 1940 bp was selected for the knockout region. The efficiency of knockout was assessed using PCR detection (Additional file 1: Fig. S1A). *Lrg1*^*−/−*^ mice were backcrossed to C75BL/6N mice for 4 generations (3 months, 18‒22 g) and raised in the SPF grade independent ventilation (IVC) laboratory of Nanchang University Laboratory. Mice were reared under conditions with a 12-h light‒dark cycle and optimal temperature and humidity. All procedures were performed to minimize the pain the animals suffered.

### Middle cerebral artery occlusion/reperfusion mouse model

Middle cerebral artery occlusion/reperfusion (MCAO/R) was conducted on mice followed by 0.5 and 1 h of occlusion and 1, 6, 12, and 24 h of reperfusion. C57BL/6 mice were anesthetized with 1.2% isoflurane. The body temperature was maintained at 37 ℃ during the ischemia period. The operation starts from the proximal external carotid stump by inserting a blunt 6-0 nylon monofilm and then pushing 9–10 mm into the internal carotid artery. After 1 h of occlusion, the nylon monofilm was withdrawn from the carotid artery, and 1, 6, 12, and 24 h of reperfusion was performed immediately afterward [14]. The same procedure was performed on the sham-operated mice, but the sutures did not enter the internal carotid artery. Animals whose blood flow dropped below 30% of pre-ischemia levels as assessed via a laser Doppler flowmeter (LDF, FLPI2, Moor, England) were used for further experiments.

### Random and blind group design for the animal experiments

The animals were assigned a random number using Excel. The experimenters were blinded to the pretreatments and data analysis.

### TTC staining for infarction volume

The brains were quickly dissected into 2-mm pieces/per section from the coronal position. The brain tissues of the group measured volume were subject to 2, 3,5-triphenyl tetrazolium chloride (TTC) staining at 37 ℃ for 20 min. The infarction volume was measured using ImageJ software (Version 1.42; Bethesda, USA). The brain tissue supplied by cerebral ischemia reperfusion appeared pale under TTC staining. The infarct volume of each stained slice was summed and expressed as a percentage.

### Measurement of brain edema via Evans blue

Two hours before mice were euthanized, Evans blue (EB) (Sigma**‒**Aldrich Co. USA) at 2% per 10 g body weight dissolved in 0.9% NaCl solution was injected into the tail vein of each mouse. After 24 h of reperfusion, brain tissues were rapidly extracted, centrifuged with 1 mL trichloroacetic acid, and homogenized at 12,000 rpm for 20 min. The absorbance of the supernatant with EB staining at 620 nm was determined by spectrophotometry. The EB concentration was determined quantitatively. The EB content was quantified as EB µg/g tissue using a standard curve.

### Neurological deficit score

The neurological deficit score is a good method to evaluate the degree of cerebral ischemia injury in animals, and the detailed method was graded based on an 18-point scale [15]. Neurological deficit sign scores were assigned to experimental animals based on an 18-point scale. Mice were tested after 24 h of reperfusion using the Longa score system as follows: 0 points, asymptomatic; 1 point, mice could not extend the opposite fore limb when lifting the tail; 2 points, mice could not rotate to the damaged side; 3 points, mice tipped uncontrollably to the opposite side; 4 points, mice could not walk normally or experienced loss of consciousness.

### Histomorphological evaluation

Histomorphological analysis was performed based on hematoxylin‒eosin (H&E) staining. After 24 h of reperfusion, mice were killed and injected with normal saline through the heart until the liquid was colorless. Then, the brain tissues were removed and immersed in a 4% formaldehyde solution in an ice bath for 24 h. Then, 5 mm brain slices were deparaffinized in xylene, rehydrated in a 100% to 70% ethanol gradient, and stained with hematoxylin‒eosin (H&E). The stained slices were subjected to gradual rinsing and imaged under a digital camera.

### Immunofluorescence staining in vivo and in vitro

Each group of brain tissues in the ischemia region was cut at a thickness of 5 mm/slice, washed with PBS (pH 7.4), incubated with cold ethanol in an ice bath for 10 min, and fixed with 10% normal goat serum, 3% bovine serum albumin, and 0.1% Triton X-100 in PBS for 1 h. Then, samples were incubated with Lrg1 (13224-1-AP, Proteintech, Chicago, USA, 1:200), CD31 (ab76533, Abcam, Cambridge, England, 1:500), IBA1 (ab178846, Abcam, Cambridge, England, 1:200), NeuN (ab177487, Abcam, Cambridge, England, 1:100), CD66b (GTX19779, GeneTex, Texas, USA, 1:1000), Claudin 11 (ab7474, Abcam, Cambridge, England, 1:200), Integrin β5 (ab177004, Abcam, Cambridge, England, 1:200), Protocadherin 9 (ab233710, Abcam, Cambridge, England, 1:100), Annexin A2 (ab216386, Abcam, Cambridge, England, 1:100), IL-6 (ab233706, Abcam, Cambridge, England, 1:50), and TNF-ɑ (ab215188, Abcam, Cambridge, England, 1:100) primary antibodies at 4 ℃ for 48 h. Brain tissues were then incubated overnight with Alexa Fluor®488-conjugated donkey anti-goat IgG H&L (Alexa Fluor®488) antibody (ab150129, Abcam, Cambridge, England, 1:1000) at 4 ℃. The cell nuclei were incubated with 4′6-diamidino-2-phenylindole (DAPI, ab104139, Abcam, Cambridge, England, 1:500) for 15 min. The immunofluorescence-stained brain tissues were observed under an Olympus FV1000 confocal microscope.

### Microvascular albumin leakage test

The skull of mice was held in the prone position, and the fur on the fixed head surface was removed to expose the skull. A circular area 5 mm in diameter was thinned using a skull drill along the cranial suture. The exposed pia mater was placed under the Lan-MC real-time vascular injury imaging system, and venules with a width of 20–40 μm were selected for observation [16]. FITC‒albumin was diluted in saline to a concentration of 1 mg/mL and infused (50 mg/kg) via the femoral vein 10 min prior to MCAO surgery. Video recording was conducted using a fluorescence-equipped microscope, and the images were analyzed using Image-Pro Plus. The video speed was set to 5 frames per second, and 9 frames were selected for each group for analysis. Fluorescence intensity was calculated by selecting areas of equal size inside and outside of the vessel, and the ratio of interstitial area fluorescence intensity (Ii) to venule fluorescence intensity (Iv) was compared with the baseline level of venules under sham operation conditions.

### Western blot and immunofluorescence staining

After the MCAO/R model was established, brain tissues were collected to extract proteins. A 40 mg protein sample collected from brain tissues in the ischemic region was separated via 12.5 % SDS-PAGE electrophoresis. The proteins were then transferred to PVDF membranes in Tris-glycine transfer buffer. The membranes were blocked with 5 % nonfat dry milk at room temperature for 2 h, incubated with anti-Lrg1 (ab178698, Abcam, Cambridge, England, 1: 1000) and GAPDH (ab8245, Abcam, Cambridge, England, 1:1000) primary antibodies in an ice bath for 24 h, and then incubated with the HRP‒conjugated secondary antibody (ab205718, Abcam, Cambridge, England, 1:2000) for 50 min. Enhanced chemiluminescence was used to display the bands, and the protein bands were captured on a biological image system (Bio-Rad, Hercules, USA).

### Single-cell suspension preparation, library construction, and next-generation sequencing

At the end of the operation, all mice were killed, and the right brain tissue was quickly removed. Under aseptic conditions, brain tissue was washed twice with precooled RPMI 1640 + 0.04% BSA medium. The clean tissue was then meticulously minced into approximately 0.5 mm^3^ fragments using surgical scissors and placed in freshly prepared digestion solution at 37 °C for 30‒60 min with intermittent agitation every 5‒10 min. The resulting cell suspension was filtered through a BD 40 μm cell strainer 1‒2 times and then centrifuged at 4 °C and 300 × *g* for 5 min. The pellet was resuspended in an appropriate volume of medium, and an equal volume of red blood cell lysis buffer (MACS, Cat^#^130-094-183) was added and mixed. Then, the sample was incubated at 4 °C for 10 min and centrifuged at 300 × *g* for 5 min. The supernatant was removed. The pellet was washed once with medium and centrifuged at 300 × *g* for 5 min. The supernatant was removed, and then the sample was resuspended in 100 μL medium. The freshly prepared single-cell suspension was adjusted to a cell concentration of 700‒1200 cell/μL, and the library was constructed according to the manufacturer's instructions for the 10 × Genomics Chromium Next GEM Single Cell 3' Reagent Kit v3.1 (Cat^#^ 000268). The constructed libraries were subjected to high-throughput sequencing on the Illumina Nova 6000 PE150 platform.

### Single-cell data processing and cell identification

Cell Ranger software (version 5.0.0) from 10 × Genomics was used to distinguish cell barcodes and allocate the sequence reads with the genome and transcriptome for each object. The resultant matrix depicted gene counts versus cells. In this study, we utilized the Scrublet tool (Version 0.2.3, https://github.com/swolock/scrublet) to predict doublets in our single-cell RNA sequencing data [17]. The rate of expected doublets was set to 0.06, and default parameters were used otherwise. We further applied quality control measures based on key metrics (the specified number of unique genes in each cell [nFeature_RNA > 500], the total detected cell number [nCount_RNA > 1000], and the percentage of mitochondrial genes in each cell [percent.mt < 25%]).

To analyze the data, we followed Seurat's pipeline (version 4.1.1, https://github.com/satijalab/seurat/) and dimensionality reduction and unsupervised clustering to perform quality control [18]. The data were normalized via the functions NormalizeData and ScaleData based on the Seurat package [18]. A total of 2,000 hypervariable genes were selected for further analysis through FindVariableFeatures, and 15 principal components were calculated for dimensionality reduction.

To correct for batch effects between groups, we utilized the BBKNN tool (Version 1.5.1, https://github.com/immunogenomics/harmony [19]. The dimensionality of clusters were reduced with the parameter "dims = 1:15, resolution = 0.3" using RunUMAP and FindClusters. Finally, we identified cells through universal markers of various cell types. Clusters that exhibited high expression of both red blood cell and small glial cell marker genes were considered doublets and excluded from analysis.

### ROGUE index

We calculated the ROGUE index using ROGUE (version 1.0, https://github.com/PaulingLiu/ROGUE). [20]. The permutation test, a widely employed statistical method for analyzing small‒sample data, was employed to assess differences in ROGUE values among distinct groups with 1,000 permutations [21].

### Calculation of cell function scores

To evaluate the functional differences among different groups of cells, we utilized Seurat's AddModuleScore function at the single‒cell level to calculate the scores of functional modules in cell clusters. We referenced the macrophage functional gene set summarized by Cheng et al. [22]. to assess the function of microglial cells and macrophages. The calculated functional modules included the M1 score and M2 score. Higher scores of various functional assessments indicate stronger corresponding functions of the cell.

### Pathway enrichment analysis

To examine the functional mechanisms of cell clusters, Gene Ontology (GO) and Kyoto Encyclopedia of Genes and Genomes (KEGG) pathway enrichment were performed on Metascape (http://metascape.org/) to identify biological pathways for a given list of genes. We introduced the first 150 differentially expressed genes with a natural logarithmic fold change (lnFC) > 0.25 for each group into Metascape for pathway enrichment analysis. This allowed us to identify enriched pathways and gain a deeper understanding of the biological processes underlying cellular functions.

### Inference of cellular differentiation trajectories

To characterize the potential lineage differentiation among cells, we employed multiple analytical methods, including CytoTRACE (version 0.3.3, https://cytotrace.stanford.edu/) and Monocle2 (version, https://github.com/kstreet13/slingshot), for cellular differentiation trajectory analysis [23]. A ﻿higher CytoTRACE score indicates that the cell is less differentiated and closer to the beginning of differentiation, whereas a ﻿lower CytoTRACE score indicates that the cell is more differentiated and closer to the end of differentiation [23]. Monocle2 was used to chart the trajectory of the potential differentiation pattern for microglial cells. Finally, we used the differential Gene Test function in Monocle2 to filter transcription factors that vary along different trajectories [24–27].

### Estimating cellular composition preference of different groups using ***R***_o/e_

In this study, we utilized the *R*_o/e_, calculated according to the method proposed by Cheng et al. [22]. The formula for *R*_o/e_ is as follows:

$$R_{{\text{o/e}}} = \frac{{{\text{Observed}}}}{{{\text{Expected}}}}.$$

Both the observed and expected values were obtained using the chisq.test function. A *R*_o/e_ value greater than 1 suggests that the cell cluster is enriched in the group, while a *R*_o/e_ value less than 1 implies that the cell cluster is not enriched in the group.

### Metabolic pathway analysis

To assess the metabolic pathway differences between different groups of cells, we downloaded 98 metabolism-related pathways from the GSEA database and estimated the metabolic pathway scores of different cell clusters using the AddModuleScore function in Seurat. For the screening of differential metabolized pathways, we used the Wilcoxon test (adjusted *p* value < 0.05) to filter out the differential metabolized pathways between the *Lrg1*^−/−^ mice and WT mice after MCAO/R, and Bonferroni correction was applied for *p* value adjustment.

### Statistical analysis

The *t* test or Wilcoxon test is employed to detect differences between two continuous groups of variables. ANOVA or the Kruskal‒Wallis test was used to examine differences between multiple groups of continuous variables. The Bonferroni method was utilized for *p* value correction. All statistical analyses were performed via R Studio (version 4.1.3) or Python (version 3.9.12). Unless otherwise specified, we defined statistical significance as a p value less than 0.05.

## Results

### Lrg1 exhibits high expression in various cell populations during cerebral ischemia‒reperfusion injury

**Fig. 1:**
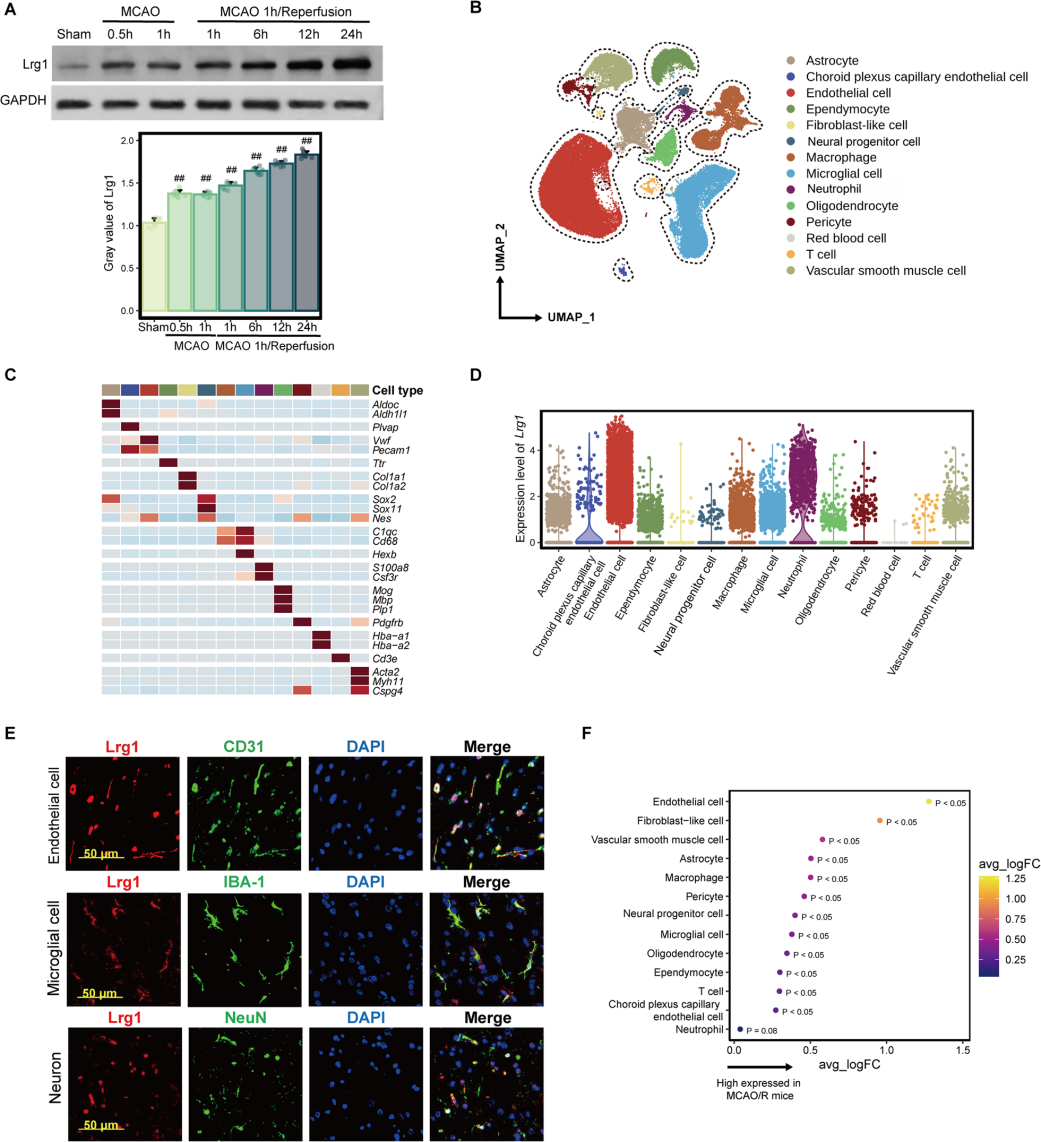
Elevated Lrg1 expression in multiple cell types in brain tissue during cerebral ischemia‒reperfusion injury. **A** Western blotting was used to determine total Lrg1 expression in brain tissues during different durations in MCAO/R mice. Mice were exposed to 0.5 and 1 h of ischemia and 1, 6, 12, and 24 h of reperfusion. Data are the mean ± SD, *n* = 8. ^##^*p* < 0.01 vs. sham group. **B** The UMAP plot presents the single-cell atlas of brain tissue after cerebral ischemia‒reperfusion injury. **C** The heatmap illustrates the expression of recognized marker genes in different cell types. **D** The violin plot displays Lrg1 expression in different cell types. **E** Immunofluorescence staining verified Lrg1 expression (red) in endothelial cells, neurons, and microglial cells in mouse brain tissues after MCAO/R. Scale bar = 50 μm. **F** The dot plot presents the differential expression analysis results of Lrg1 in various cell types between the MCAO/R and sham groups. An average log fold change (avg_logFC) value > 0 indicates that Lrg1 is expressed at higher levels in the MCAO/R group than in the sham group

Western blotting was applied to examine the change in Lrg1 expression in the brain during cerebral ischemia‒reperfusion injury. As shown in Fig. 1A, during cerebral occlusion, Lrg1 was upregulated in the ischemic brains, and expression gradually increased with longer reperfusion injury. High Lrg1 expression continued until 24 h after reperfusion (Fig. 1A).

To investigate the cell distribution of Lrg1, we analyzed published scRNA-seq data, which contain scRNA-seq data from the same left cerebral hemispheres from three sham-operated mice as well as three mice with tMCAO (transient middle cerebral artery occlusion) operated 24 h after ischemia reperfusion [9]. After stringent quality control, we obtained 58,077 high-quality cells. Subsequently, we used marker genes to classify these cells into different cell types (Fig. 1B, C). We identified 14 cell types, including oligodendrocytes, endothelial cells, neural progenitor cells, microglial cells, macrophages, astrocytes, vascular smooth muscle cells, ependymocytes, T cells, pericytes, neutrophils, choroid plexus capillary endothelial cells, fibroblast-like cells, and red blood cells. Next, we characterized the expression pattern of Lrg1 in different cell types. Lrg1 expression was observed in almost all the cell types of the cerebral ischemia‒reperfusion injury brain, including astrocytes, choroid plexus capillary endothelial cells, endothelial cells, ependymocytes, fibroblast-like cells, neural progenitor cells, macrophages, microglial cells, neurons, oligodendrocytes, pericytes, T cells, neutrophils, and vascular smooth muscle cells (Fig. 1D). We verified Lrg1 protein expression in several cell types using immunofluorescence and observed that the Lrg1 protein was expressed in endothelial cells, microglial cells and neutrophils (Fig. 1E, Additional file 1: Fig. S1C). Simultaneously, we employed immunofluorescence to detect the expression of Lrg1 in neuronal cells (Fig. 1E, Additional file 1: Fig. S1C).

Moreover, we computed the differential expression of Lrg1 in brain tissue after MCAO/R and sham-operated brain tissue. Our findings revealed that *Lrg1* was upregulated in various cell types, including endothelial cells, fibroblast-like cells, vascular smooth muscle cells, astrocytes, and macrophages after MCAO/R (Fig. 1F). Overall, we observed that compared to the sham-operated group, many types of cells in cerebral ischemia‒reperfusion injury brain tissue showed increased *Lrg1* expression, suggesting that Lrg1 plays an important role in modulation of the cerebral ischemia‒reperfusion injury process and may be related to many cell types in the brain.

### Conventional *Lrg1* knockout (*Lrg1*^−/−^) attenuates infarct volume, water content, and neurological deficits induced by cerebral ischemia‒reperfusion injury

**Fig. 2:**
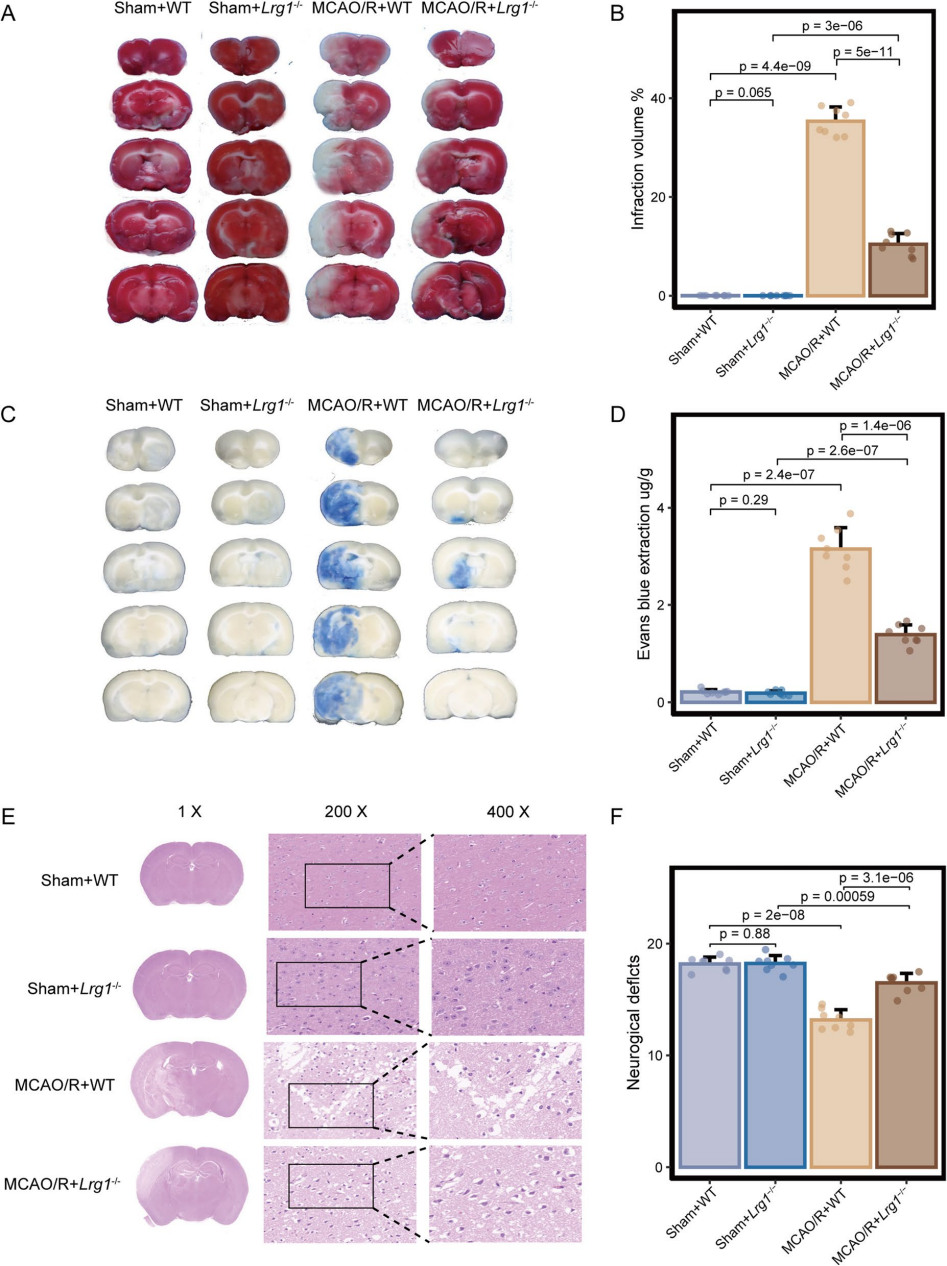
Effects of *Lrg1* knockout on brain tissue damage in cerebral ischemia‒reperfusion mice. Mice were exposed to 1 h of ischemia and 24 h of reperfusion. **A**, **B** Gross slice figures of MCAO/R mouse brains stained with 2,3,5**‒**triphenyl tetrazolium chloride. The brain appeared red based on the interaction between TTC and dehydrogenase in the no infarction area, and the color faded to white in the no infarction area. Error bar graph showing the results of TTC staining in different groups. Data are expressed as the mean ± SD, *n* = 8. **C**, **D** Gross appearance of brains exposed to 1 h of ischemia and 24 h of reperfusion was observed based on Evans blue staining. Error bar graph showing the results of Evans blue staining in different groups. Data are expressed as the mean ± SD, *n* = 8. **E** H&E staining reveals morphology of brain tissues of MCAO/R mice based on light microscopic assessment. The damaged brain tissues exhibited white interspaces, pyknotic nuclei, appeared holes, and signs of bleeding. Scale bar = 1 × ; 200 × ; 400 × . **F** Measurement of neurological deficit scores of MCAO/R mice. Error bar graph showing the results of neurological deficit score in different groups. Data are expressed as the mean ± SD, *n* = 8

To validate the role of Lrg1 as a target in cerebral ischemia‒reperfusion injury, we compared the cerebral infarct volume, water content, and neurological deficits of *Lrg1*^−/−^ mice and WT mice after MCAO/R (Fig. 2). The deletion efficiency of Lrg1 was previously verified in *Lrg1*^−/−^ mice via western blotting (Additional file 1: Fig. S1B). We observed that knockout of Lrg1 led to significant reductions in cerebral infarct volume (Fig. 2A, B), cerebral edema (Fig. 2C, D), and neurological deficits (Fig. 2F) after cerebral ischemia‒reperfusion injury. In normal brain tissues, immunohistochemical staining revealed positive purple‒red staining. The cells were full with distinct nuclei and cytoplasm, and the brain tissue was compact. Immunohistochemical staining of frozen sections revealed vacuolation and concentrated and dead neurons in ischemic brain tissues, whereas the morphology was significantly improved in *Lrg1*^−/−^ mice injured by MCAO/R (Fig. 2E). In summary, our findings demonstrate that *Lrg1* knockout confers protection to brain tissue after cerebral ischemia‒reperfusion injury.

### Single-cell RNA sequencing reveals significant alterations in the transcriptional profiles of various cell types in brain tissue after cerebral ischemia‒reperfusion injury upon *Lrg1* knockout

**Fig. 3:**
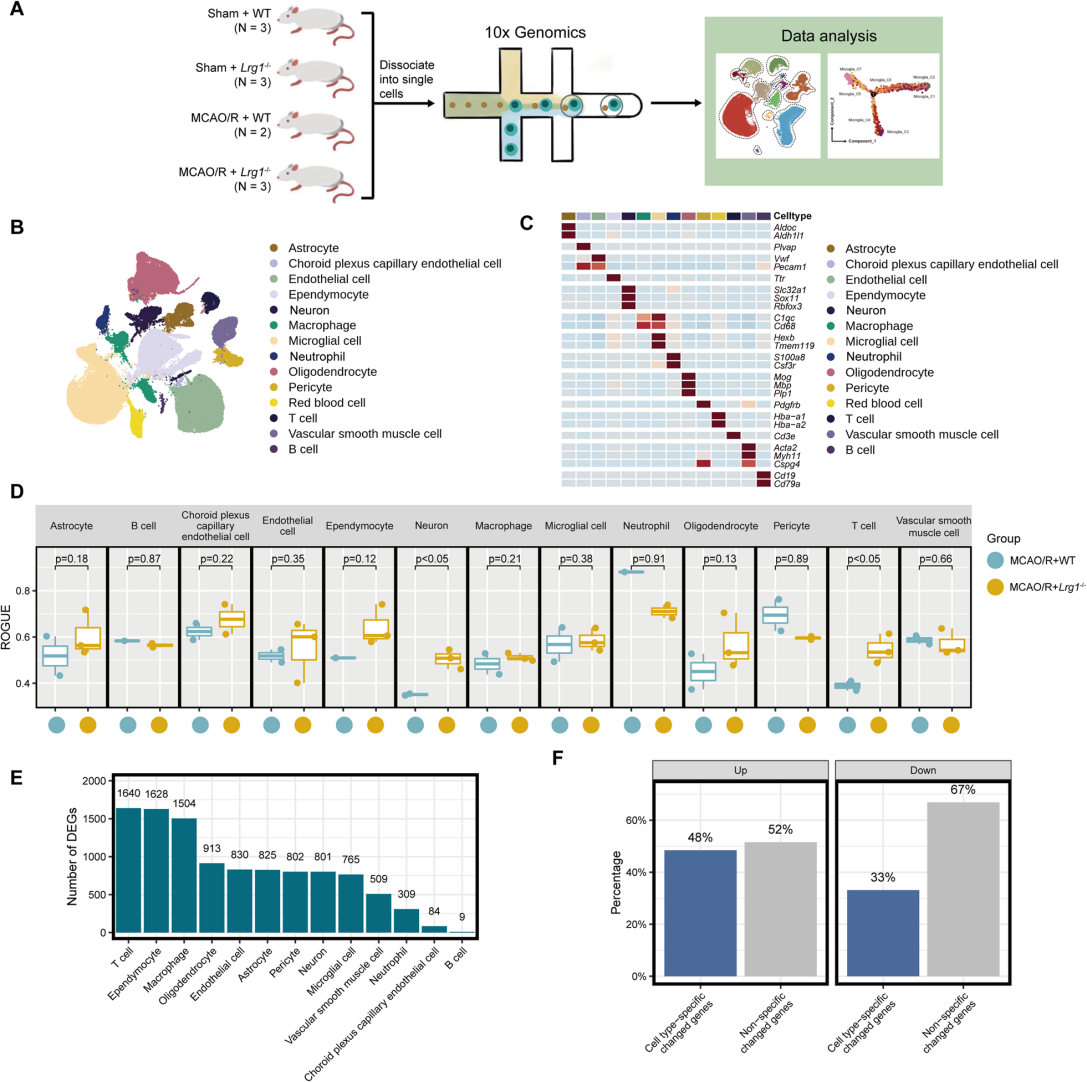
Single-cell transcriptomic data demonstrate that the impact of *Lrg1* knockout on various cellular components in brain tissue of cerebral ischemia‒reperfusion mice. **A** A schematic representation of the experimental design employed in this study is depicted. **B** UMAP plots of 99,991 cells from 11 mice, including 14 cell types. **C** Heatmap of gene expression across different cell types. **D** Boxplot displaying cell purity for cell types. **E** Bar graph showing the number of differentially expressed genes for different cell types. **F** Bar graph showing the proportion of genes with unique changes in a single cell type among differentially upregulated or downregulated genes in MCAO/R + *Lrg1*^−/−^ mice compared to MCAO/R + WT mice

Multiple cell types showed high Lrg1 expression levels in the brains of mice with cerebral ischemia‒reperfusion injury (Fig. 1D, F), suggesting that the protective effect of *Lrg1* knockout on brain tissue after cerebral ischemia‒reperfusion injury may result from its action on multiple cell types. To investigate the impact of *Lrg1* knockout on various cells following cerebral ischemia‒reperfusion injury, we conducted single-cell RNA sequencing (scRNA-seq) on cells obtained from the brains of WT sham-operated mice (sham + WT group), *Lrg1*^−/−^ sham-operated mice (sham + *Lrg1*^−/−^ group), WT mice after MCAO/R (MCAO/R + WT group), and *Lrg1*^−/−^ mice after MCAO/R (MCAO/R + *Lrg1*^−/−^ group) (Fig. 3A). After strict quality control filtering (Additional file 2: Fig. S2A, B), a total of 99,991 cells were identified from 11 mice (including 29,709 cells in the sham + WT group, 13,938 cells in the sham + *Lrg1*^−/−^ group, 20,572 cells in the MCAO/R + WT group, and 35,772 cells in the MCAO/R + *Lrg1*^−/−^ group). The cell populations were defined via unsupervised clustering analysis on the obtained cells, and each cluster was identified as a specific cell type using recognized marker genes (Fig. 3B). In total, we obtained 14 types of cells, including ependymocytes, neurons, endothelial cells, vascular smooth muscle cells, microglial cells, red blood cells, B cells, oligodendrocytes, astrocytes, pericytes, T cells, choroid plexus capillary endothelial cells, macrophages, and neutrophils (Fig. 3B, C).

Next, we explored the effects of *Lrg1* knockout on different cell types. First, using ROGUE, we observed that compared to the cell composition in WT mice, the cell composition in *Lrg1*^−/−^ mice showed lower expression heterogeneity after MCAO/R (Fig. 3D). Furthermore, we identified the differentially expressed genes (DEGs) in different cell populations in WT and *Lrg1*^−/−^ mouse brains. As shown in Fig. 3E, we observed that many cell types showed a large number of DEGs (average of 817 DEGs), with the largest number observed in T cells, ependymocytes, macrophages, oligodendrocytes, and endothelial cells (Fig. 3E). B cells and choroid plexus capillary endothelial cells had fewer DEGs (Fig. 3E). To confirm whether the transcriptional profile changes after *Lrg1* knockout are consistent across different cell types, we investigated the overlap of differentially expressed genes (DEGs) in various cell types. As shown in Fig. 3F, we observed that a significant proportion of DEGs (52% in upregulated DEGs and 33% in downregulated DEGs of the *Lrg1*^*−/−*^ group after MCAO/R) were altered exclusively in a single cell type. These findings suggest that different cell types exhibits distinct changes in gene expression profiles following *Lrg1* knockout, and the changes induced by Lrg1 differ across different cell populations. Therefore, we focused on deciphering the expression profile changes in different cell types. Overall, our results suggest that *Lrg1* knockout changes the expression profiles of different cell types and that its effects on different cell compositions vary.

### Conventional knockout of Lrg1 restricts the BBB dysfunction after cerebral ischemia‒reperfusion injury

**Fig. 4:**
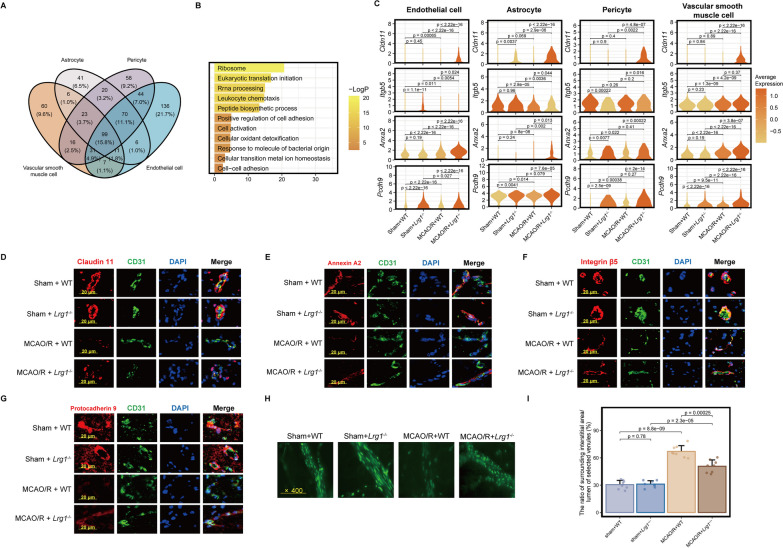
*Lrg1* knockout reduces BBB dysfunction after cerebral ischemia‒reperfusion injury in mice. **A** Venn diagram illustrating the overlapping differentially expressed genes in BBB constituent cells (upregulated in brain tissue of *Lrg1*^−/−^ mice after cerebral ischemia‒reperfusion injury compared to WT mice). **B** Pathway enrichment analysis results of the 99 overlapping differentially expressed genes of BBB constituent cells. **C** Violin plots displaying the expression of intercellular adhesion molecules in BBB constituent cells of different groups and colored according to the standardized expression median. **D**–**G** Representative immunofluorescence images showing the protein expression of Claudin 11, Annexin A2, Integrin β5, and Protocadherin 9 in the brain tissues of mice from different groups. Scale bar = 20 μm. **H** Microvascular albumin leakage test showing the change in albumin leakage in the venules of the brain tissues from different mouse groups. **I** Error bar graph showing the results of the microvascular albumin leakage test in different groups. Data are expressed as the mean ± SD, *n* = 8. Scale bar = 400 ×

Prior studies have shown that following cerebral ischemia‒reperfusion injury, the connections between components of the blood‒brain barrier become disrupted, leading to a series of symptoms, such as brain edema [8, 28]. Through differential gene analysis (Fig. 3E), we observed that the blood‒brain barrier cell composition had a greater number of DEGs after *Lrg1* knockout. This finding prompted us to investigate the effect of *Lrg1* knockout on the BBB. We extracted the constituent cells of the BBB, including astrocytes, vascular smooth muscle cells, pericytes, and endothelial cells, and used a Venn diagram to visualize the overlap of upregulated genes after *Lrg1* knockout (Fig. 4A). The Venn diagram results suggested that there are many nonoverlapping DEGs among the different blood‒brain barrier cell types after *Lrg1* knockout, indicating that these cells exhibit different changes (Fig. 4A). In addition, we observed 99 genes that showed consistent changes in these cell types. Furthermore, we performed pathway enrichment analysis on these 99 genes. The pathway enrichment analysis revealed that these associated genes include ribosome, positive regulation of cell adhesion, and cellular oxidant detoxification (Fig. 4B). A weakened connection between BBB components can lead to the development of brain edema after cerebral ischemia‒reperfusion injury. Moreover, we observed that the expression levels of some cell adhesion molecules, such as claudin 11, integrin β5, annexin A2, and protocadherin 9, were increased in the BBB cells of *Lrg1*^−/−^ mice compared with those of WT mice after MCAO/R (Fig. 4C) [29–31]. Subsequently, we used immunofluorescence to examine the protein expression of cell adhesion-related proteins in endothelial cells in brain tissues of different groups. As shown in Fig. 4D–G, compared to WT mice, *Lrg1*^−/−^ mice exhibited increased protein levels of cell adhesion-related molecules, including Cldn11 (Fig. 4D), Anxa2 (Fig. 4E), Pcdh9 (Fig. 4F), and Itgb5 (Fig. 4G), in endothelial cells after cerebral ischemia‒reperfusion injury. These results suggest that Lrg1 can regulate the expression of these cell adhesion-related molecules in endothelial cells.

Furthermore, by injecting FITC-labeled albumin through the tail vein, we measured the permeability of cerebral venule in different group. According to the statistical data of fluorescence intensity, the ratio of surrounding interstitial area (Ii) fluorescence intensity to the fluorescence intensity of the lumen of selected venules (Iv) in the same area increased after MCAO/R (Ii/Iv: sham + WT: 30.56 ± 4.75%, MCAO/R + WT: 67.01 ± 6.38%), indicating that vascular damage led to increased permeability (Fig. 4H, I). However, *Lrg1* knockout attenuated this leakage and decreased the proportion of fluorescent leakage to levels significantly lower than that observed in the MCAO/R + WT group (Ii/Iv: MCAO/R + *Lrg1*^−/−^: 50.80 ± 6.88%, MCAO/R + WT: 67.01 ± 6.38%, Fig. 4H, I). The findings presented herein suggest that *Lrg1* knockout attenuate the disruption of the BBB following cerebral ischemia‒reperfusion injury in cerebral tissue.

### *Lrg1* knockout shifts microglial cells and macrophages from a proinflammatory state to an anti-inflammatory and tissue repair-promoting state after cerebral ischemia‒reperfusion injury

**Fig. 5:**
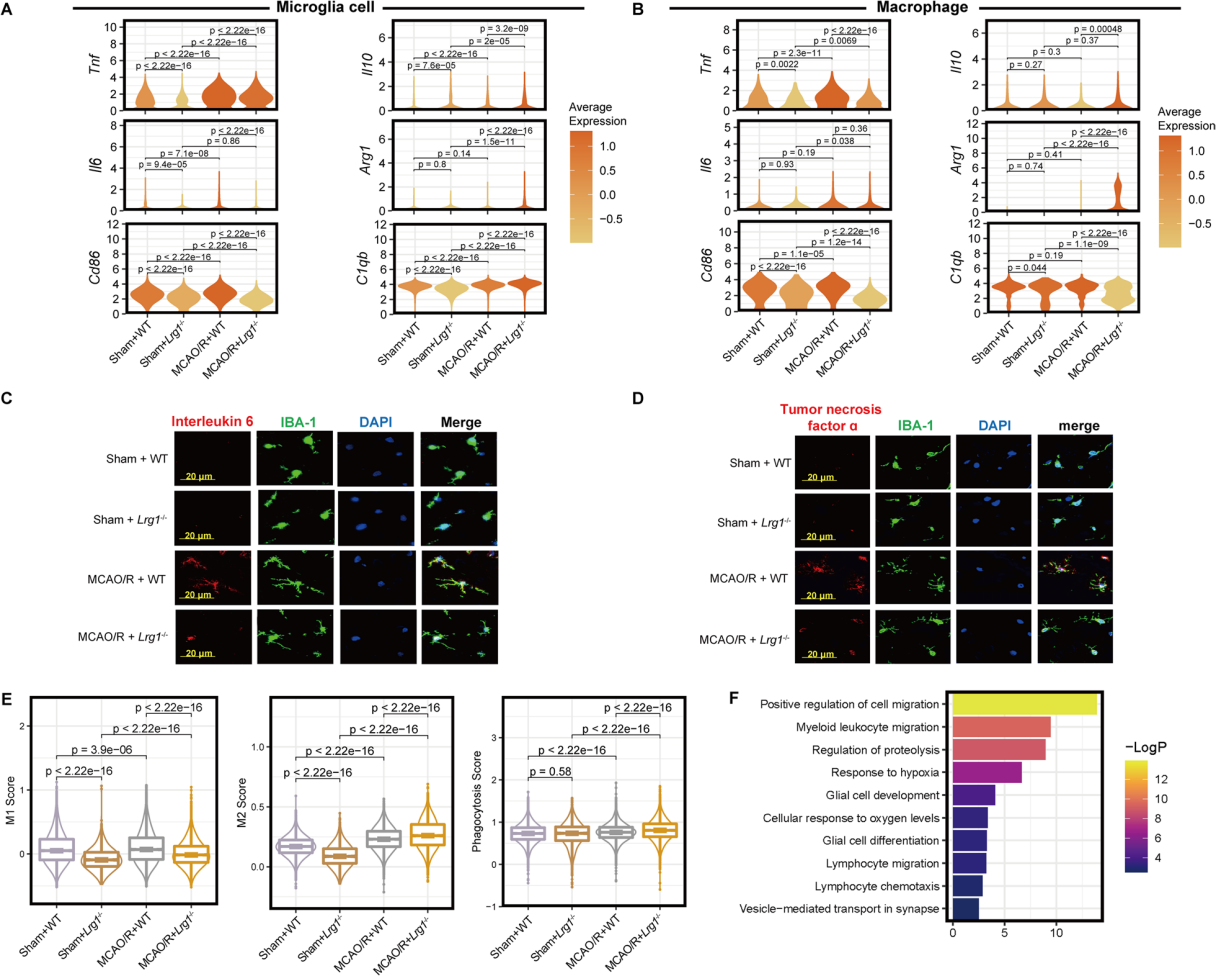
*Lrg1* knockout alters the functional state of microglial cells and macrophages after cerebral ischemia‒reperfusion injury in mice. **A** Violin plot showing the expression of functional molecules in microglial cells of different groups. The coloring is based on the standardized median expression of each group. **B** Violin plot exhibiting the expression of functional molecules in macrophages of different groups. The coloring is based on the standardized median expression of each group. **C** Representative images showing interleukin 6 protein expression in the brain tissues of mice from different groups. Scale bar = 20 μm. **D** Representative images showing tumor necrosis factor α protein expression in the brain tissues of mice from different groups. Scale bar = 20 μm. **E** Box plot showing the functional state score of microglial cells from different groups. **F** Pathway enrichment analysis of upregulated differentially expressed genes in microglial cells in brain tissues of *Lrg1*^−/−^ mice after MCAO/R compared with WT mice after MCAO/R

Microglial cells are acknowledged as macrophages resident central nervous system (CNS), that play roles in sustaining and nourishing the CNS as well as immune surveillance [32, 33]. Through differential gene analysis (Fig. 3E), we observed that microglial cells and macrophages had a greater number of DEGs after *Lrg1* knockout, prompting us to investigate the effect of *Lrg1* knockout on these cells. Our differential gene analysis revealed that microglial cells and macrophages in WT mice exhibited higher expression levels of proinflammatory molecules (interleukin 6, tumor necrosis factor ɑ, Fig. 5A, B) and M1-type macrophage markers (*Cd86*, Fig. 5A, B) compared with *Lrg1*^−/−^ mice after MCAO/R [34, 35]. In contrast, microglial cells and macrophages in the MCAO/R + *Lrg1*^−/−^ group had higher expression levels of anti-inflammatory molecules (*Il10*, Fig. 5A and B) and tissue repair-promoting molecules (*Arg1*, Fig. 5A, B) compared with the MCAO/R + WT group [36]. Using immunofluorescence, we observed that microglial cells in *Lrg1*^−/−^ mice exhibited lower protein expression levels of proinflammatory cytokines, such as interleukin 6 and tumor necrosis factor ɑ, than those in WT mice (Fig. 5C, D). We also observed that microglial cells in *Lrg1*^−/−^ mice exhibited higher expression of molecules associated with phagocytic function (*C1qb*), but macrophages in the WT mice did not show this characteristic (Fig. 5A, B) [37].

Furthermore, we used various functional scores to measure the functional states of microglial cells and macrophages in different groups (Fig. 5E). Functional scores showed that microglial cells and macrophages in *Lrg1*^−/−^ mice were more M2 biased than those in WT mice after MCAO/R. The microglial cells of *Lrg1*^−/−^ mice also had stronger phagocytic ability than the microglial cells of WT mice after MCAO/R (Fig. 5E). The findings indicate that *Lrg1* knockout leads to a reduction in the proinflammatory functions of microglial cells and macrophages along with an increase in their ability to suppress inflammation and promote tissue repair.

In addition, we performed pathway enrichment analysis on the upregulated DEGs in microglial cells of *Lrg1*^−/−^ mice. The results showed that microglial cells of *Lrg1*^−/−^ mice were associated with various biological processes, such as positive regulatory mechanisms of cell migration, response to hypoxia, and glial cell development (Fig. 5F). These results suggest that *Lrg1* knockout may affect biological processes, such as movement, response to hypoxia, and differentiation of microglial cells.

### *Lrg1* knockout impacts microglial cell differentiation after cerebral ischemia‒reperfusion injury

**Fig. 6:**
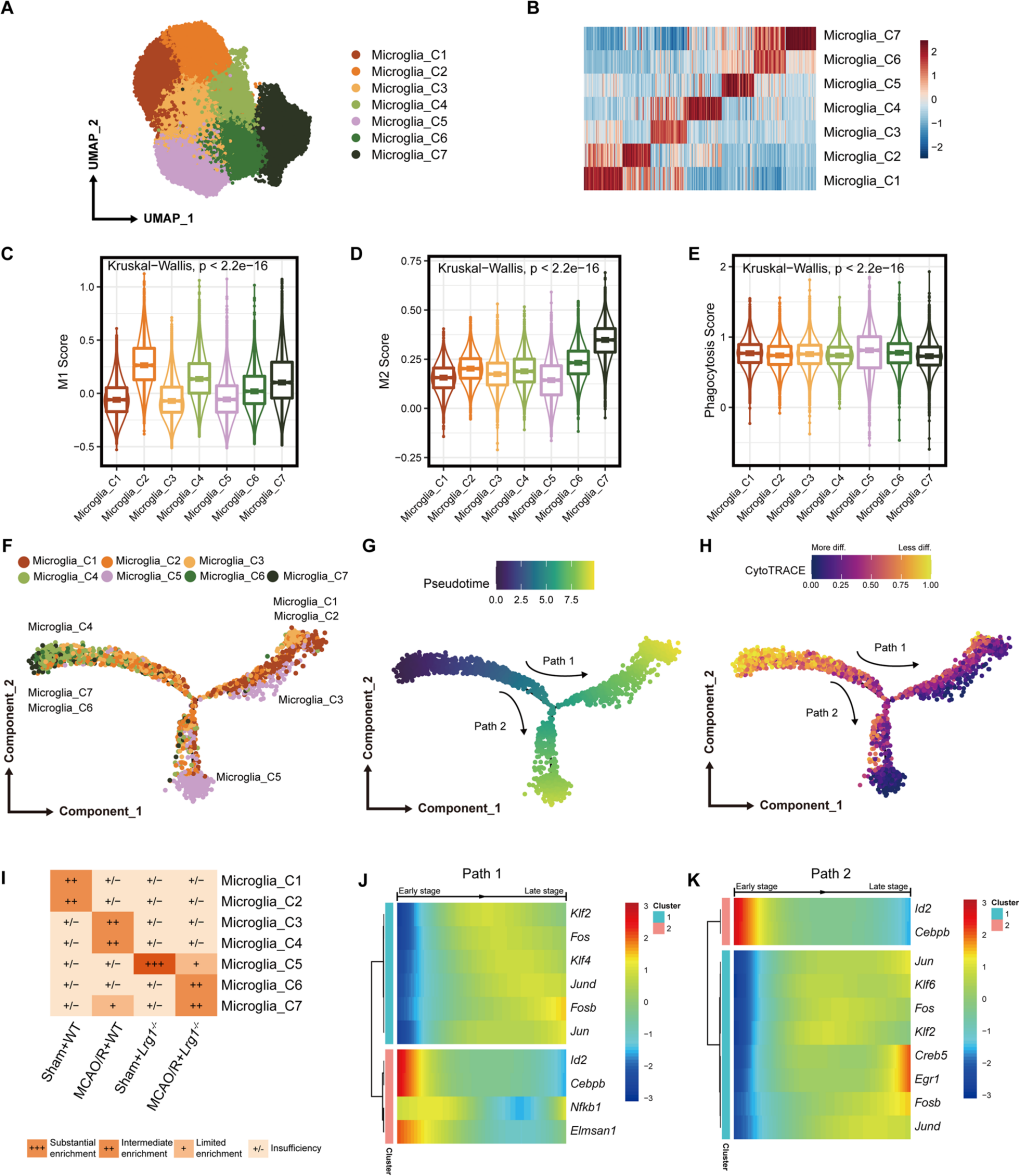
*Lrg1* knockout affects microglial cell maturation after cerebral ischemia‒reperfusion injury in mice. **A** The UMAP plot illustrates the clustering results of microglial cells in brain tissue after MCAO/R. **B** The heatmap depicts the expression of the top 50 differentially expressed genes in distinct microglia subpopulations. **C** The box plot demonstrates the M1 score in microglial cells across different clusters. **D** The box plot exhibits the M2 score in microglial cells across different clusters. **E** The box plot displays the phagocytosis scores in microglia across different clusters. **F** The dot plot demonstrates the distribution of different clusters in the differentiation trajectory plot of microglial cells in Sham + WT group constructed using monocle2. **G** The dot plot illustrates the differentiation trajectories of microglial subpopulations in Sham + WT group drawn by monocle2. The direction of arrows indicates the direction of differentiation and development. **H** The dot plot illustrates the differentiation trajectories of microglial subpopulations in Sham + WT group drawn by CytoTRACE. The direction of arrows indicates the direction of differentiation and development. **I** Heatmap visualizations depicted the enrichment profiles of distinct cell clusters (rows) across diverse groups (columns). The coloration within the cells corresponds to the *R*_o/e_ values, denoting the extent of cell cluster enrichment as delineated below: Insufficiency: *R*_o/e_  ≤ 1; Limited Enrichment: 1 < * R*_o/e_  ≤ 1.5; Intermediate Enrichment: 1.5 < * R*_o/e_  ≤ 3; Substantial Enrichment: *R*_o/e_  > 3. **J** The heatmap illustrates the transcription factors of Path 1 that change with the trajectory in Sham + WT group. Different TF clusters signify TFs with varying expression patterns along the trajectory. **K** The heatmap demonstrates the transcription factors of Path 2 that change with the trajectory in Sham + WT group. Different TF clusters represent TFs with distinct expression patterns along the trajectory

Based on pathway enrichment analysis showing that *Lrg1* knockout may impact microglial cell differentiation (Fig. 5F), we analyzed microglial cell differentiation trajectories. First, we performed unsupervised clustering analysis on the obtained microglial cells. In total, we obtained 30,973 microglial cells that were clustered into 7 clusters (Fig. 6A). Significant differences in the expression profiles of different clusters were demonstrated through heatmap visualization of the signature genes of different clusters (Fig. 6B). To explore the functions of different microglial subtypes, we first calculated various functional scores for each cluster. We observed that the functional states of different microglial clusters were significantly different (Fig. 6C‒E). Microglia_C2, Microglia_C4, Microglia_C6, and Microglia_C7 had higher M1 scores, whereas Microglia_C1, Microglia_C3, and Microglia_C5 had lower M1 scores (Fig. 6C). Additionally, we observed that Microglia_C6 and Microglia_C7 had higher M2 scores, whereas Microglia_C1, Microglia_C2, Microglia_C3, Microglia_C4, and Microglia_C5 had lower M2 scores (Fig. 6D). These results demonstrated that the phenotype of microglial cells in brain tissue after MCAO/R is more complex and does not simply consist of two polarization groups, M1 and M2. Overall, Microglia_C2 and Microglia_C4 were more biased toward the M1 type, whereas Microglia_C6 and Microglia_C7 were more biased toward the M2 type. The results of phagocytic scoring indicated that Microglia_C5 had the highest phagocytic score, suggesting that it has the strongest phagocytic ability (Fig. 6E).

Subsequently, we sought to investigate the differentiation trajectory of microglial cells. We calculated the differentiation trajectories of microglial cells separately in different groups using Monocle 2 and CytoTRACE. Combining the results, we observed that microglial cells in the brain tissue followed two differentiation trajectories: one trajectory from Microglia_C4, Microglia_C6, and Microglia_C7 to Microglia_C1, Microglia_C2 and Microglia_C3 (Path 1, Fig. 6F–H and Additional file 4: Fig. S4) and another trajectory from Microglia_C4, Microglia_C6, and Microglia_C7 to Microglia_C5 (Path 2, Fig. 6F–H and Additional file 4: Fig. S4).

Furthermore, we quantified the distribution of different microglial subtypes across various groups by calculating Ro/e values (Fig. 6I). We observed distinct subtype distributions among microglial cells in different groups. Specifically, Microglia_C1 and Microglia_C2 were predominantly enriched in the Sham + WT group (Fig. 6I). Microglial cells from Microglia_C3, Microglia_C4, and Microglia_C7 were enriched in the MCAO/R + WT group (Fig. 6I). Microglia_C5 was predominantly enriched in the Sham + *Lrg1*^*−/−*^ group, whereas the MCAO/R + *Lrg1*^*−/−*^ group exhibited a greater abundance of Microglia_C5, Microglia_C6, and Microglia_C7 (Fig. 6I). In conjunction with the previous findings, these results suggest alterations in the differentiation states of microglial cells following MCAO/R or *Lrg1* knockout. In summary, our findings suggest that MCAO/R and Lrg1 may modulate microglial differentiation.

Transcription factors (TFs) shape different cell phenotypes and regulate cell differentiation processes [38]. Therefore, we screened for differentially expressed TFs along the microglial differentiation trajectory. We conducted transcription factor screening in microglial cells from the Sham + WT group (Fig. 6J, K). Our results showed that in Path 1, *Id2*, *Cebpb* and other TFs expression decreased with differentiation, whereas the expression of *Fos*, *Klf4*, and other TFs increased with differentiation (Fig. 6J). In Path 2, our results showed that *Id2* and *Cebpb* expression decreased with differentiation, whereas that of *Jun*, *Klf6*, *Egr1*, and other TFs increased with differentiation (Fig. 6K). Furthermore, we validated the majority of these transcription factors exhibiting similar patterns of change in two trajectories within the other three groups (Additional file 5: Fig. S5). These TFs may influence the differentiation of microglia, and Lrg1 may also impact microglial cells by modulating the expression of these TFs.

### *Lrg1* knockout reduces neuron and oligodendrocyte death after cerebral ischemia‒reperfusion injury

A total of 1782 neurons were obtained in our study. The enriched gene sets of upregulated genes in neurons of WT mice after MCAO/R (compared with neurons of *Lrg1*^−/−^ mice after MCAO/R) showed that *Lrg1* knockout was associated with various processes, such as nucleosome assembly, adjustment of function in processes of apoptosis signal, and modulation of neuron death (Additional file 6: Fig. S6A), indicating that neurons of WT mice after MCAO/R may be more susceptible to cell death compared to neurons of *Lrg1*^−/−^ mice after MCAO/R. Jin et al. reported that Lrg1 overexpression enhances neuronal apoptosis and autophagy after cerebral ischemia‒reperfusion injury, which is consistent with our observations [39].

Oligodendrocytes wrap axons in the central nervous system to form the myelin sheath structure, which is essential for neuronal function [40]. Previous studies revealed that after cerebral ischemia‒reperfusion injury, the death of oligodendrocytes leads to a reduction in the myelin sheath structure [41, 42]. A total of 12,547 oligodendrocytes were obtained in our study. The enriched gene sets of upregulated genes in oligodendrocytes of WT mice after MCAO/R (compared with oligodendrocytes of *Lrg1*^−/−^ mice after MCAO/R) showed that *Lrg1* knockout was associated with biological processes, such as rRNA processing, positive regulation of cell death, and apoptosis (Additional file 6: Fig. S6B), suggesting that *Lrg1* knockout may reduce oligodendrocyte death and protect the myelin sheath structure.

Concomitantly, based on differential gene calculations, we found that vascular smooth muscle cells from *Lrg1*^−/−^ mice expressed higher levels of *Ngf* than those from WT mice after MCAO/R, which could promote neuronal repair following MCAO/R injury (Additional file 6: Fig. S6C).

### *Lrg1* knockout alters the metabolic status of multiple cell populations after cerebral ischemia‒reperfusion injury

**Fig. 7:**
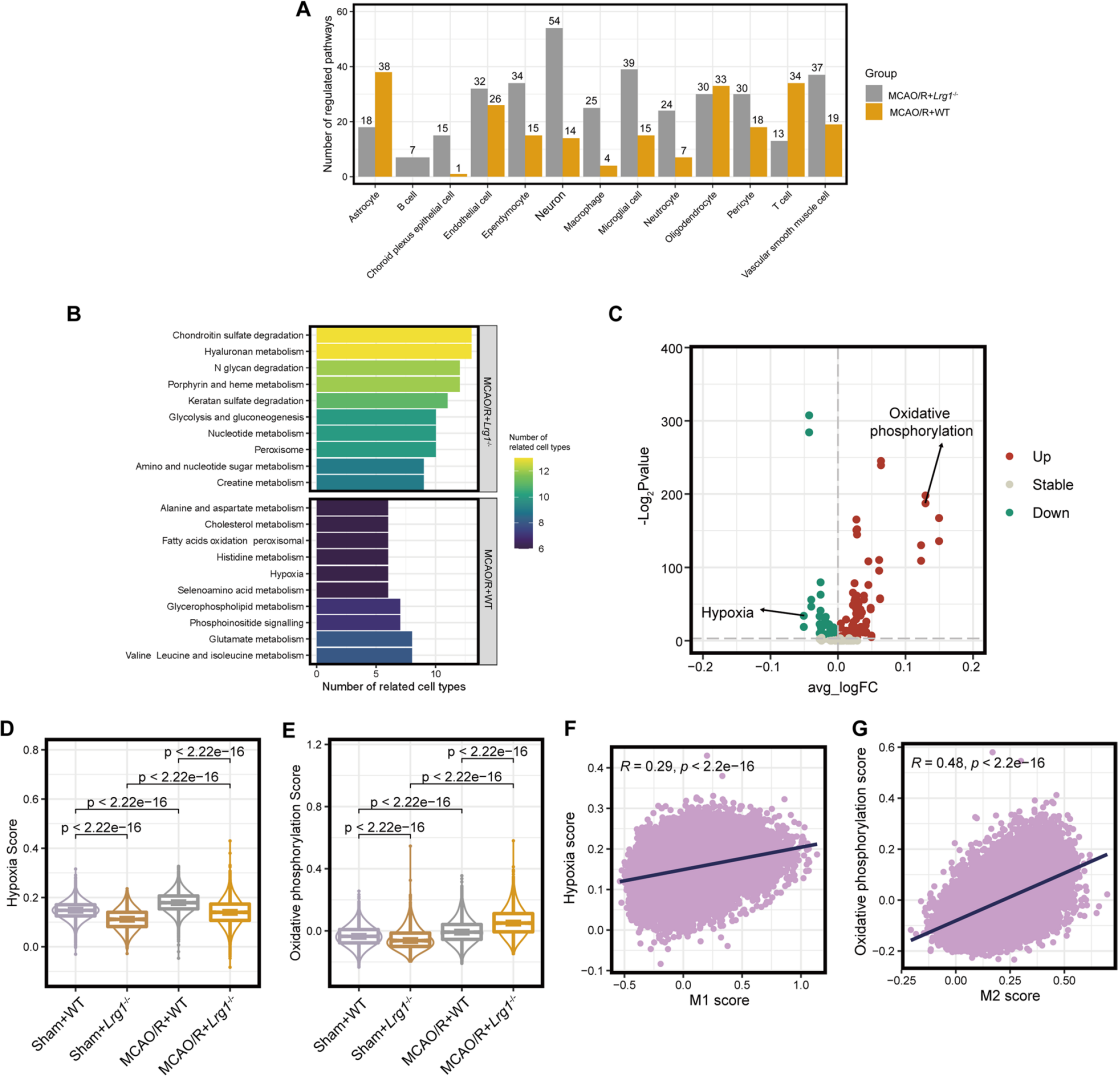
*Lrg1* knockout alters the metabolic status of multiple cell populations after cerebral ischemia‒reperfusion injury in mice. **A** The bar chart displays the differences in metabolic pathways between various cell populations in different groups. **B** The bar chart presents the common differentially regulated metabolic pathways in various cell populations between different groups. **C** The volcano plot depicts the differentially regulated metabolic pathways in microglial cells between different groups. **D** The box plot shows the results of oxidative phosphorylation scores in microglial cells from different groups. **E** The box plot displays the results of hypoxia scores in microglial cells from different groups. **F** The dot plot demonstrates the correlation between hypoxia scores and M1 scores in microglial cells. **G** The dot plot illustrates the correlation between oxidative phosphorylation scores and M2 scores in microglial cells

Various metabolic processes are associated with cerebral ischemia‒reperfusion injury-induced brain tissue damage [43–45]. Therefore, we estimated the metabolic status of various cell compositions in different groups based on their expression profiles. We then calculated the number of differential metabolic pathways for different cell types (adjusted p value < 0.05). As shown in Fig. 7A, we observed that the differential metabolic status of different cell types varied. In WT mice and *Lrg1*^−/−^ mice, multiple metabolic pathways were differentially expressed in most cell types, such as neurons, vascular smooth muscle cells, and microglial cells, whereas B cells showed fewer differential metabolic pathways (Fig. 7A). These results suggest that *Lrg1* knockout may alter the metabolic status of multiple cell populations in the brain after cerebral ischemia‒reperfusion injury. Furthermore, we identified the common differential metabolic pathways in multiple cell types (Fig. 7B). Our results revealed that several pathways, such as chondroitin sulfate degradation, hyaluronan metabolism, and N glycan degradation, were significantly upregulated in multiple cell types of WT mice (Fig. 7B). Current studies have shown that the upregulation of chondroitin sulfate proteoglycans is associated with the glial reaction, resulting in the formation of inhibitory scars that impede axonal growth and network reorganization of damaged neurons [46]. In contrast, we observed that hypoxia was upregulated in most cell types in WT mice. Hypoxia may induce various types of cell damage after cerebral ischemia‒reperfusion injury [47]. In summary, our results indicate that *Lrg1* knockout alters the metabolic status of multiple cell components.

Prior investigations have substantiated the influence of diverse metabolic pathways on the polarization status of microglial cells in neurological diseases [48, 49]. Based on our previous results, we found that *Lrg1* knockout could affect the state of microglia and various metabolic pathways in the cerebral ischemia‒reperfusion injury process (Figs. 5B, 7A). We believe that there may be a potential correlation between these pathways, so we examined the differential metabolic pathways in microglia of cells from WT mice and *Lrg1*^−/−^ mice. We observed that the oxidative phosphorylation pathway was stronger in microglial cells of the MCAO/R + *Lrg1*^−/−^ group compared with the MCAO/R + WT group, whereas hypoxia was stronger in microglial cells from WT mice (Fig. 7C–E). Mitochondrial oxidative phosphorylation is inhibited in activated M1 macrophages, preventing their transition to an M2 phenotype [50]. Hypoxia promotes M1 polarization of macrophages [48, 51]. However, their effects on microglial cells and brain tissue after cerebral ischemia‒reperfusion injury are unclear. This notion prompted us to evaluate the association between these pathway scores in microglia. Our calculations showed that hypoxia was significantly positively correlated with the M1 score (Pearson correlation coefficient = 0.29, Fig. 7F), whereas the oxidative phosphorylation score was significantly positively correlated with the M2 score in microglia (Pearson correlation coefficient = 0.48, Fig. 7G). Overall, we believe that oxidative phosphorylation and hypoxia may influence the polarization state of microglial cells in cerebral ischemia‒reperfusion injury.

## Discussion

**Fig. 8:**
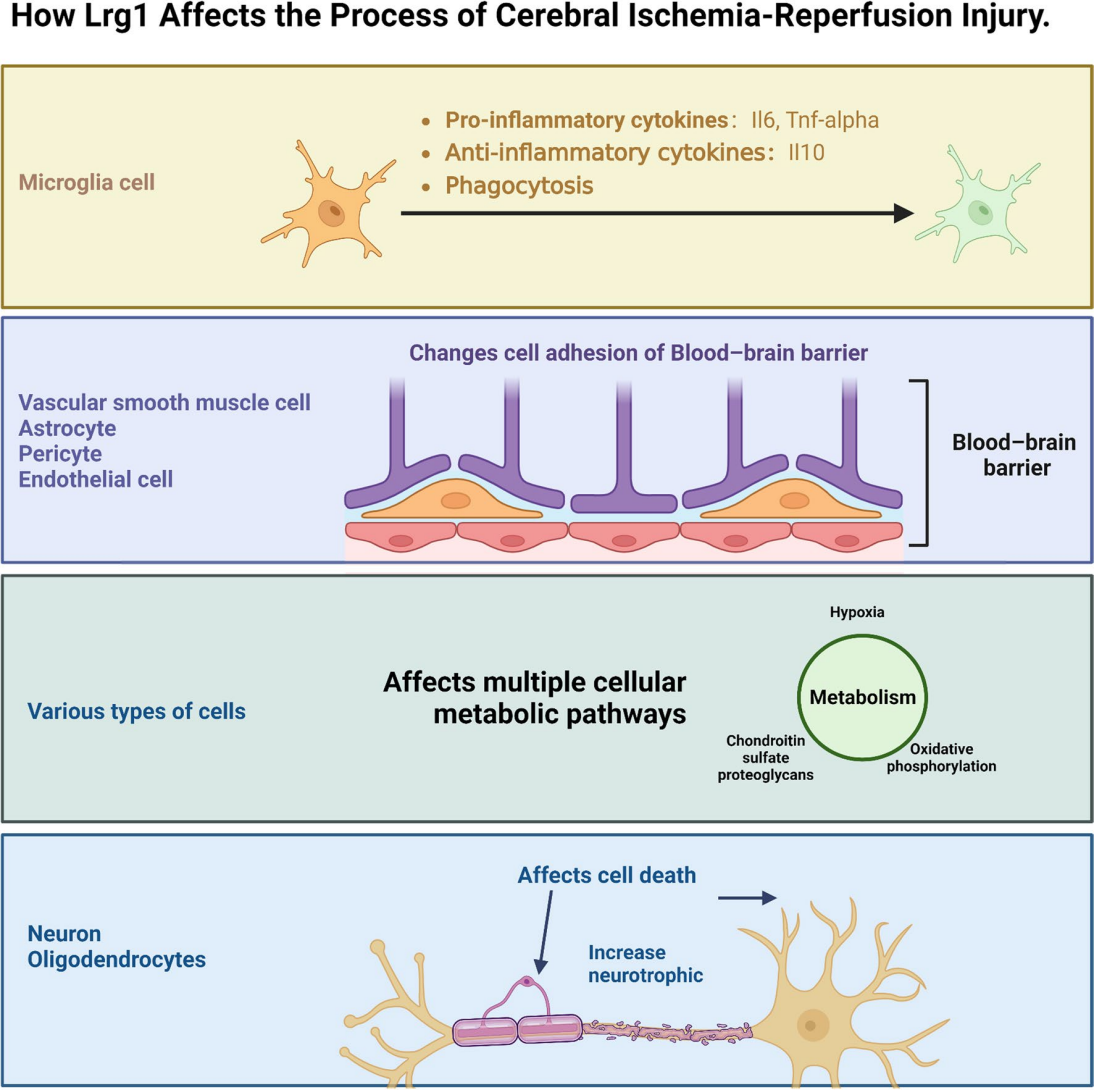
Schematic diagram of the mechanism by which Lrg1 affects the process of cerebral ischemia‒reperfusion injury in mice

Cerebral ischemia‒reperfusion injury is a major cause of global stroke mortality and inflicts significant damage to human health. Despite current therapeutic interventions, the effectiveness of treatments for cerebral ischemia‒reperfusion injury remains limited. Therefore, innovative and effective treatments to treat cerebral ischemia‒reperfusion injury are urgently needed. Our study demonstrates that *Lrg1* knockout effectively protects brain tissue from cerebral ischemia‒reperfusion injury. Using scRNA-seq data from gene knockout mice, we identified potential mechanisms involving multiple brain tissue cell components. The data indicated that knockout of *Lrg1* had no significant difference on cerebral infarct volume, cerebral edema, and neurological deficits compared with WT mice under the sham‒operated condition (Fig. 2). Our findings show that *Lrg1* knockout reduce cell junction damage in the BBB after cerebral ischemia‒reperfusion injury and promotes the transition of microglia from a proinflammatory state to a tissue repair‒promoting role. Additionally, it reduces the death of neurons and oligodendrocytes. Further analysis revealed that *Lrg1* knockout may affect the differentiation and metabolic state of microglia, altering their functional status (Fig. 8).

Here, we used scRNA‒seq to decipher the cellular atlas of different brain tissues after cerebral ischemia‒reperfusion injury. We identified 14 types of cells, including ependymocytes, neurons, vascular smooth muscle cells, red blood cells, oligodendrocytes, astrocytes, pericytes, T cells, choroid plexus capillary endothelial cells, macrophages, microglial cells, B cells, endothelial cells, and neutrophils. Our results were largely consistent with previous findings, but we obtained a higher number of cells, which allowed us to identify B cells that were not previously reported by other researchers.

A recent study demonstrated that Lrg1 upregulation is associated with increased brain infarct volume, neuronal apoptosis, and enhanced autophagy, exacerbating cerebral ischemia‒reperfusion injury [11, 12]. Consistent with previous findings, Lrg1 expression continues to increase in ischemia‒reperfusion brain tissues with prolonged reperfusion time. However, it remains unclear in which specific cells Lrg1 expression is elevated after cerebral ischemia‒reperfusion injury. Here, this research indicates that Lrg1 is upregulated in multiple cell types during cerebral ischemia‒reperfusion injury, suggesting its involvement in disease progression across various cellular components. Thus, inhibiting Lrg1 expression may provide a multifaceted approach to protect brain tissue from injury. Furthermore, we have demonstrated experimentally that *Lrg1* knockout helps to attenuate cerebral ischemia‒reperfusion injury. These findings highlight the potential of Lrg1 as a therapeutic target for cerebral ischemia‒reperfusion injury.

Furthermore, we investigated the changes in various cell types in brain tissue after cerebral ischemia‒reperfusion injury following *Lrg1* knockout to elucidate the role of Lrg1 in cerebral ischemia‒reperfusion injury. We employed single-cell RNA sequencing to explore potential mechanisms. We measured the overlap of differentially expressed genes (DEGs) in various cell components to assess whether *Lrg1* knockout results in similar changes in gene expression profiles. Our results showed limited overlap of DEGs in different cell components, indicating that different cell components exhibit distinct gene expression changes after *Lrg1* knockout. Therefore, we will focus on analyzing the gene expression profile changes in different cells in future studies. Current studies have shown that after cerebral ischemia‒reperfusion injury, both oligodendrocytes and neurons undergo cell death [52]. White matter (region comprising neuronal axons) injury, which accounts for approximately half of the volume of stroke, is a central cause of nerve function impairments [53]. However, white matter injury is often overlooked in research. Due to their larger size, some neurons are difficult to capture using single-cell sequencing. Nevertheless, certain studies have managed to isolate a small number of these neurons through this method [54, 55]. Possibly benefiting from a larger number of cells and samples, we have also acquired a small population of neural cells here for the purpose of dissecting neural cell characteristics. Previous research has also suggested that Lrg1 overexpression increases neuron apoptosis, which is consistent with our findings. Moreover, our results suggest that *Lrg1* knockout may alleviate oligodendrocyte apoptosis after cerebral ischemia‒reperfusion injury. These findings suggest that downregulation of Lrg1 may potentially reduce demyelination following cerebral ischemia‒reperfusion injury and subsequently mitigate the occurrence of postcerebral ischemia‒reperfusion injury seizures. However, our findings are based on computational analysis and should be confirmed in the future.

The tight junctions of the BBB possess critical functions in cerebral ischemia‒reperfusion injury. Based on our observations, we found that *Lrg1* knockout could ameliorate brain edema after cerebral ischemia‒reperfusion injury. Previous studies have suggested that Lrg1 can affect the functional state of endothelial cells, but its relationship with the tight junctions of endothelial cells and other components of the blood‒brain barrier remains unclear [56]. To the best of our knowledge, we are the first to report that *Lrg1* knockout can block the leakage of the BBB, promoting BBB integrity after cerebral ischemia‒reperfusion injury. We observed that in WT mice, some cell adhesion molecules may be upregulated after MCAO/R (Fig. 4C). We postulate that this may represent a reparative mechanism in response to the breakdown of the BBB, where some cell adhesion molecules are mildly upregulated to facilitate BBB repair. A similar phenomenon was observed in microglial cells, as shown in Fig. 5A and B, where some molecules that aid in post‒MCAO/R injury repair were also upregulated. We believe that *Lrg1* knockout may regulate the expression of critical junction molecules, such as *Cldn11* and *Anxa2*, affecting their protein expression and ultimately preserving BBB stability after cerebral ischemia‒reperfusion injury and reducing brain edema.

The involvement of microglial cells and macrophages in the progression of brain disease following cerebral ischemia‒reperfusion injury has been established in previous studies [6, 57, 58]. Proinflammatory M1-type microglia and macrophages promote cerebral ischemia‒reperfusion injury progression, whereas anti-inflammatory M2-type microglia and macrophages alleviate cerebral ischemia‒reperfusion injury progression and also phagocytosing dead cells to aid in recovery following cerebral ischemia‒reperfusion injury [59, 60]. To date, no studies have examined the relationship between Lrg1 and the functional state of microglial cells and macrophages. Here, we used scRNA‒seq to analyze the effects of *Lrg1* knockout on the functional state of microglial cells following cerebral ischemia‒reperfusion injury. Specifically, compared to WT microglial cells, microglia lacking Lrg1 showed decreased expression of proinflammatory molecules (such as IL-6 and TNF-ɑ) but increased expression of anti-inflammatory molecules (such as IL-10) and enhanced phagocytic function. Therefore, the protective effect of *Lrg1* knockout in cerebral ischemia‒reperfusion injury may be due to alterations in the microglial functional state. Furthermore, we explored how *Lrg1* knockout affects the microglial functional state. On the one hand, our results suggest that *Lrg1* knockout may influence the microglial differentiation trajectory. Previous studies have shown that after brain injury, the microenvironment's small glial cells can be classified into M1 and M2 phenotypes, each playing pro-inflammatory and anti-inflammatory roles, respectively [61]. Our research findings revealed that the status of microglial cells in cerebral ischemia‒reperfusion injury brain tissue is more complex and cannot be solely encompassed by the M1 and M2 polarization states, which is consistent with previous studies. Moreover, our results suggest that *Lrg1* knockout may influence the differentiation trajectory of microglial cells. This action could represent the mechanism underlying the change in the microglial functional state observed following *Lrg1* knockout. On the other hand, we found that Lrg1 may affect the microglial metabolic state, specifically by enhancing oxidative phosphorylation and reducing hypoxia. Oxidative phosphorylation may enhance the M2 phenotype of microglial cells in some neurodegenerative diseases, whereas hypoxia can promote the M1 phenotype of macrophages. Here, we established a link between microglial M1 and M2 states and their score status in relation to oxidative phosphorylation and hypoxia in cerebral ischemia‒reperfusion injury using computational methods. We propose that Lrg1 may switch microglial functional states by altering their oxidative phosphorylation and hypoxic states, but more research is needed to support this conclusion, as our findings are based exclusively on computational results. Additionally, existing research has indicated that the transport of metabolites between cells serves as a means of cellular communication. Our findings suggest that upon knocking out *Lrg1*, various cells undergo alterations in metabolic pathways, hinting at the possibility of intercellular communication through metabolic products, such as lactate shuttling, in this process. However, due to data limitations, we are currently unable to make a definitive judgment on this aspect, which necessitates further exploration using additional omics data, including metabolomics, and metabolic experiments.

Apart from these findings, our study still has several limitations. For example, we only elucidated the impact of *Lrg1* knockout on a subset of cells, and further analysis is warranted for other cell types, such as T cells and B cells. For instance, research has highlighted the potential significance of Lrg1 in modulating the function of neutrophils [62, 63]. Our investigations also detected the expression of Lrg1 in neutrophils, which aligns with previous studies. However, upon knocking out *Lrg1*, we discerned that the differential genes in neutrophils were fewer in comparison to other cells. Given the limited number of neutrophils we obtained, we believe it is premature to conclusively determine the influence of Lrg1 on neutrophils at this juncture. The interplay between Lrg1 and neutrophils during ischemia‒reperfusion injury warrants further exploration in future studies.

In addition, our data only examined the short-term effects of *Lrg1* knockout on postcerebral ischemia‒reperfusion injury brain tissue at one hour, and the long-term effects of *Lrg1* knockout on postcerebral ischemia‒reperfusion injury brain tissue remain unclear. Thus, more experiments are needed to further explore the influence of Lrg1 on cerebral ischemia‒reperfusion injury.

In summary, our investigation has elucidated how Lrg1 modulates the process of cerebral ischemia‒reperfusion injury by altering the functional states of multiple cellular components of the brain. These alterations include enhancing BBB connectivity, transforming the states of microglia and macrophages, and reducing neuron and oligodendrocyte death, as shown in Fig. 8. Our findings suggest Lrg1 as a promising therapeutic target for cerebral ischemia‒reperfusion injury; however, further experimentation and efforts are needed to refine our understanding of its precise mechanisms in this process.

## Supplementary Information

**Additional file 1: Figure S1.** Genotypic characterization of *Lrg1* knockout mice. Tail genomic DNA of *Lrg1*^*−/−*^ mice was used for PCR genotyping according to the corresponding primers. Lrg1 expression in brain tissues from *Lrg1*^*−/−*^ mice was detected by western blotting. Data are expressed as the mean ± SD, n = 3, MCAO/R + *Lrg1*^−/−^ vs. MCAO/R + WT. Immunofluorescence staining verified Lrg1 expression (red) in neutrophils in mouse brain tissues after MCAO/R. Scale bar = 50 μm.

**Additional file 2: Figure S2.** Basic information of single‒cell RNA‒seq data.

**Additional file 3: Figure S3.** Functional status scores of macrophages in different groups.

**Additional file 4: Figure S4.** Differentiation trajectory analysis of microglia in the different groups. A Differentiation trajectory analysis of microglia in the MCAO/R + WT Group. Scatter plots depict distinct clusters (left), Pseudotime scores computed by Monocle 2 (center), and CytoTREACE scores (right), respectively. B Differentiation trajectory analysis of microglia in the Sham + *Lrg1*^*−/−*^ Group. Scatter plots depict distinct clusters (left), Pseudotime scores computed by Monocle 2 (center), and CytoTREACE scores (right), respectively. C Differentiation trajectory analysis of microglia in the MCAO/R + *Lrg1*^*−/−*^ Group. Scatter plots depict distinct clusters (left), Pseudotime scores computed by Monocle 2 (center), and CytoTREACE scores (right), respectively.

**Additional file 5: Figure S5.** The transcription factors associated with microglial cell differentiation. A The expression profiles of the selected transcription factors, varying with pseudotime, were validated along two trajectories in the MCAO/R + WT Group. B Validation of the selected transcription factors' expression profiles, changing with pseudotime, along two trajectories in the Sham + *Lrg1*^*−/−*^ Group. C Examination of the expression patterns of the selected transcription factors, modulated by pseudotime, along two trajectories in the MCAO/R + *Lrg1*^*−/−*^ Group.

**Additional file 6: Figure S6.** Functional enrichment analysis of differentially expressed genes upregulated in neurons and astrocytes of WT mice compared to *Lrg1*^−/−^ mice. A. Functional enrichment analysis results of differentially expressed genes upregulated in neurons of WT mice compared to *Lrg1*^−/−^ mice after cerebral ischemia‒reperfusion injury. B. Functional enrichment analysis results of differentially expressed genes upregulated in oligodendrocytes of WT mice compared to *Lrg1*^−/−^ mice after cerebral ischemia‒reperfusion injury. C. Dot plot displaying the expression of Ngf in various cell types of brain tissues from different groups.

## Data Availability

The data reported in this study are available at NCBI GEO (https://www.ncbi.nlm.nih.gov/geo/query/acc.cgi?acc=GSE245386).

## Abbreviations

BBB: Blood‒brain barrier
BSA: Bovine serum albumin
CNS: Central nervous system
DEGs: Differentially expressed genes
EB: Evans blue
GSEA database: Gene Set Enrichment Analysis
H&E: Hematoxylin‒eosin
HRP: Horseradish peroxidase
IL-6: Interleukin-6
Lrg1: Leucine-rich alpha-2-glycoprotein 1
*Lrg1*^*−*^^*/*^^*−*^ mice: *Lrg1* Knockout mice
MCAO/R: Middle cerebral artery occlusion/reperfusion
PCA: Principal component analysis
PCR: Polymerase chain reaction
PVDF: Polyvinylidene fluoride
scRNA-seq: Single-cell RNA sequencing
TFs: Transcription factors
TGF-β: Transforming growth factor-β
TNF-α: Tumor necrosis factor-alpha
TTC: Triphenyl tetrazolium chloride
UMAP: Uniform Manifold Approximation and Projection
WT: Wild-type
