# Correction: Single-cell RNA sequencing unveils Lrg1’s role in cerebral ischemia‒reperfusion injury by modulating various cells

**DOI:** 10.1186/s12974-025-03610-4

**Published:** 2025-11-14

**Authors:** Zhaohui Ruan, Guosheng Cao, Yisong Qian, Longsheng Fu, Jinfang Hu, Tiantian Xu, Yaoqi Wu, Yanni Lv

**Affiliations:** 1 Department of Pharmacy, The First Affiliated Hospital of Nanchang University, Nanchang, China; 2 College of Pharmacy, Hubei University of Chinese Medicine, Wuhan, China; 3 School of Clinical Medicine, Nanchang University, Nanchang, China

**Correction: Journal of Neuroinflammation 20, 285 (2023)**

**https://doi.org/10.1186/s12974-023-02941-4**

In this article [1], the author would like to correct the following two errors.

1. One figure in Fig. 2E has been misused in the published version of this article.

Incorrect Figure 2

**Figure 1:**
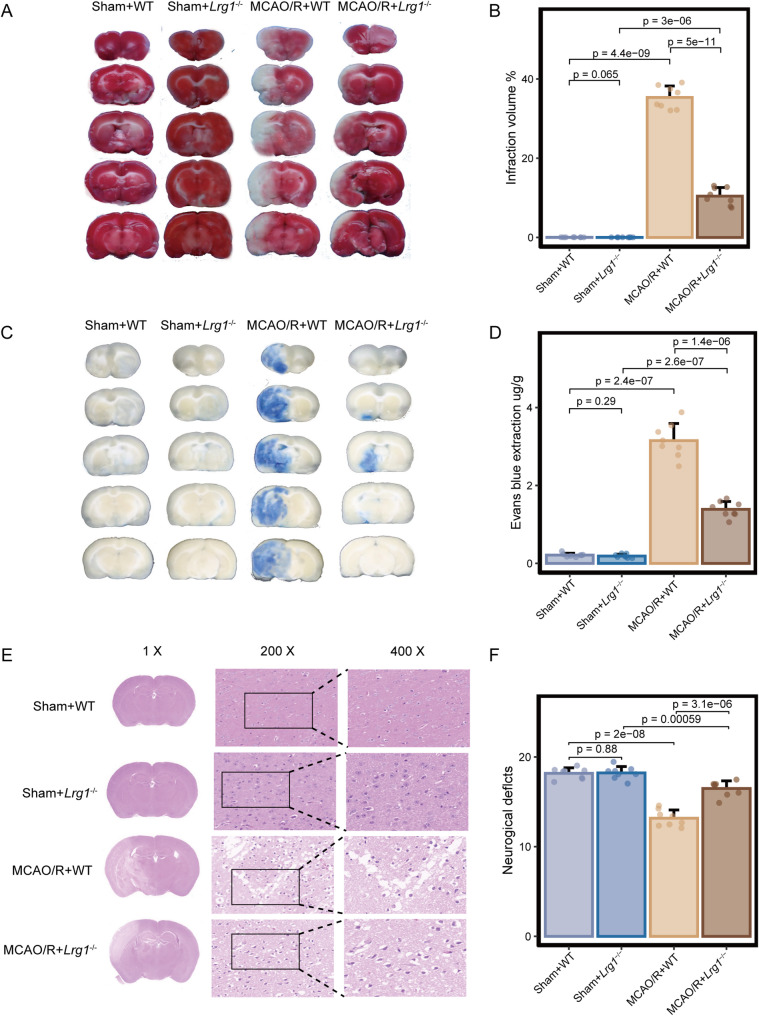
Effects of Lrg1 knockout on brain tissue damage in cerebral ischemia‒reperfusion mice. Mice were exposed to 1 h of ischemia and 24 h of reperfusion. **A**, **B** Gross slice figures of MCAO/R mouse brains stained with 2,3,5‒triphenyl tetrazolium chloride. The brain appeared red based on the interaction between TTC and dehydrogenase in the no infarction area, and the color faded to white in the no infarction area. Error bar graph showing the results of TTC staining in different groups. Data are expressed as the mean ± SD, *n* = 8. **C**, **D** Gross appearance of brains exposed to 1 h of ischemia and 24 h of reperfusion was observed based on Evans blue staining. Error bar graph showing the results of Evans blue staining in different groups. Data are expressed as the mean ± SD, *n* = 8. **E** H&E staining reveals morphology of brain tissues of MCAO/R mice based on light microscopic assessment. The damaged brain tissues exhibited white interspaces, pyknotic nuclei, appeared holes, and signs of bleeding. Scale bar = 1 × ; 200 × ; 400 ×. **F** Measurement of neurological deficit scores of MCAO/R mice. Error bar graph showing the results of neurological deficit score in different groups. Data are expressed as the mean ± SD, *n* = 8

Correct Figure 2

**Figure 2:**
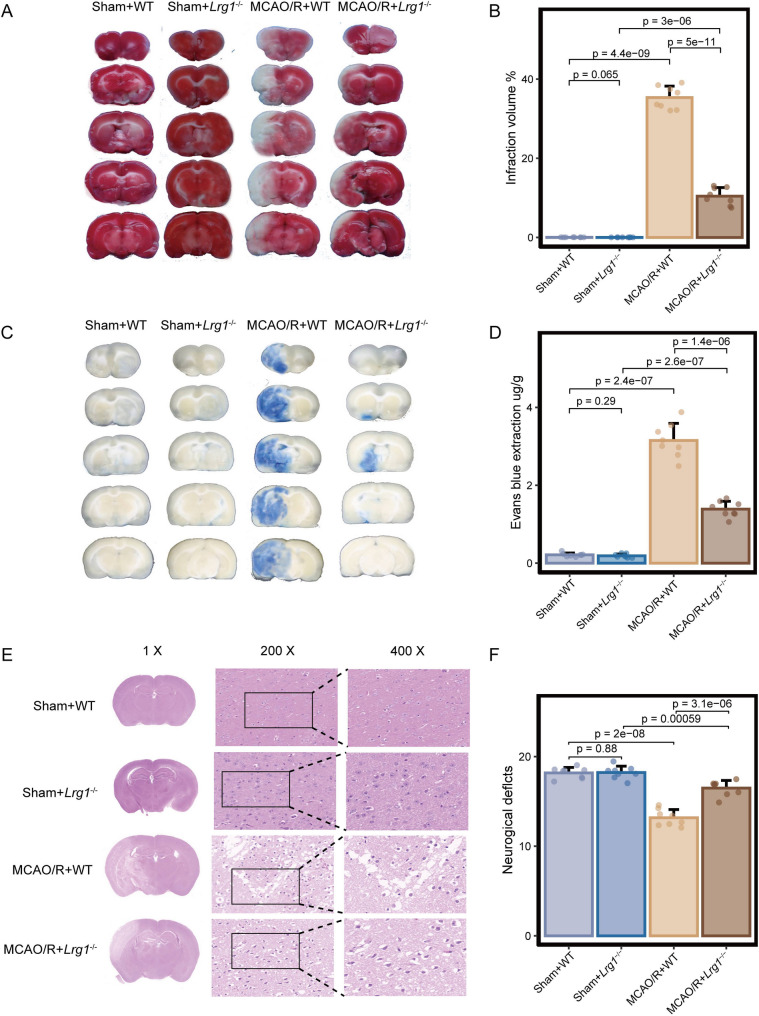
Effects of Lrg1 knockout on brain tissue damage in cerebral ischemia‒reperfusion mice. Mice were exposed to 1 h of ischemia and 24 h of reperfusion. **A**, **B** Gross slice figures of MCAO/R mouse brains stained with 2,3,5‒triphenyl tetrazolium chloride. The brain appeared red based on the interaction between TTC and dehydrogenase in the no infarction area, and the color faded to white in the no infarction area. Error bar graph showing the results of TTC staining in different groups. Data are expressed as the mean ± SD, *n* = 8. **C**, **D** Gross appearance of brains exposed to 1 h of ischemia and 24 h of reperfusion was observed based on Evans blue staining. Error bar graph showing the results of Evans blue staining in different groups. Data are expressed as the mean ± SD, *n* = 8. **E** H&E staining reveals morphology of brain tissues of MCAO/R mice based on light microscopic assessment. The damaged brain tissues exhibited white interspaces, pyknotic nuclei, appeared holes, and signs of bleeding. Scale bar = 1 × ; 200 × ; 400 ×. **F** Measurement of neurological deficit scores of MCAO/R mice. Error bar graph showing the results of neurological deficit score in different groups. Data are expressed as the mean ± SD, *n* = 8

2. In the Methods part, “Single‑cell data processing and cell identifcation”.

The sentence “We further applied quality control measures based on key metrics (the specifed number of unique genes in each cell [nFeature_RNA > 500], **the total detected cell number** [nCount_RNA > 1000], and the percentage of mitochondrial genes in each cell [percent.mt < 25%])”, the description of “the total detected cell number” should be corrected as “the total UMI count per cell”.
