# Supplementary figures and images for: Single-cell RNA sequencing unveils Lrg1's role in cerebral ischemia‒reperfusion injury by modulating various cells
Source: J Neuroinflammation. 2023 Nov 30;20:285. doi: 10.1186/s12974-023-02941-4 (PMC10687904; doi:10.1186/s12974-023-02941-4)

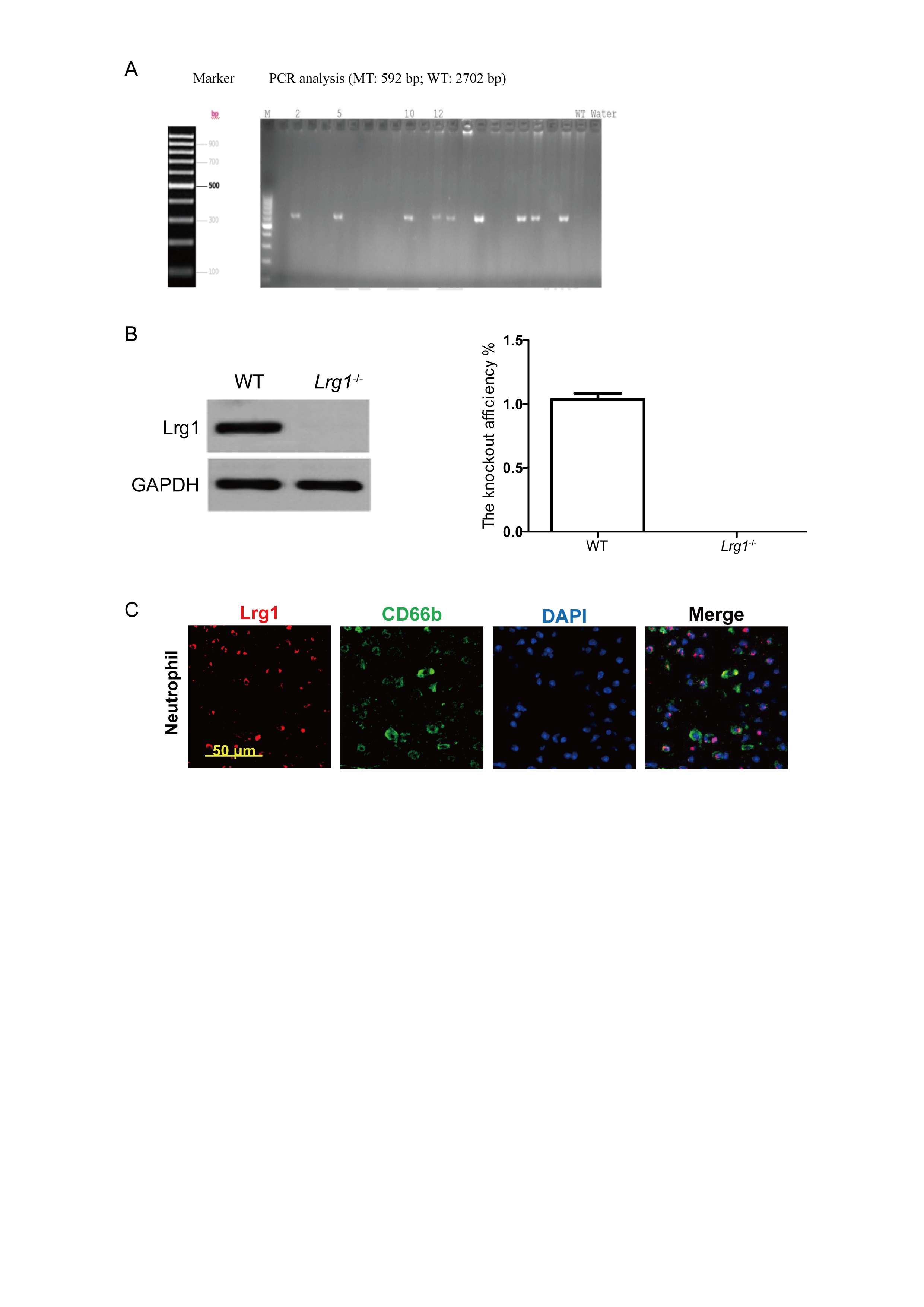

Supplement: Supplementary file 1 — Additional file 1: Figure S1. Genotypic characterization of Lrg1 knockout mice. Tail genomic DNA of Lrg1−/− mice was used for PCR genotyping according to the corresponding primers. Lrg1 expression in brain tissues from Lrg1−/− mice was detected by western blotting. Data are expressed as the mean ± SD, n = 3, MCAO/R + Lrg1−/− vs. MCAO/R + WT. Immunofluorescence staining verified Lrg1 expression (red) in neutrophils in mouse brain tissues after MCAO/R. Scale bar = 50 μm. [file 12974_2023_2941_MOESM1_ESM.png]

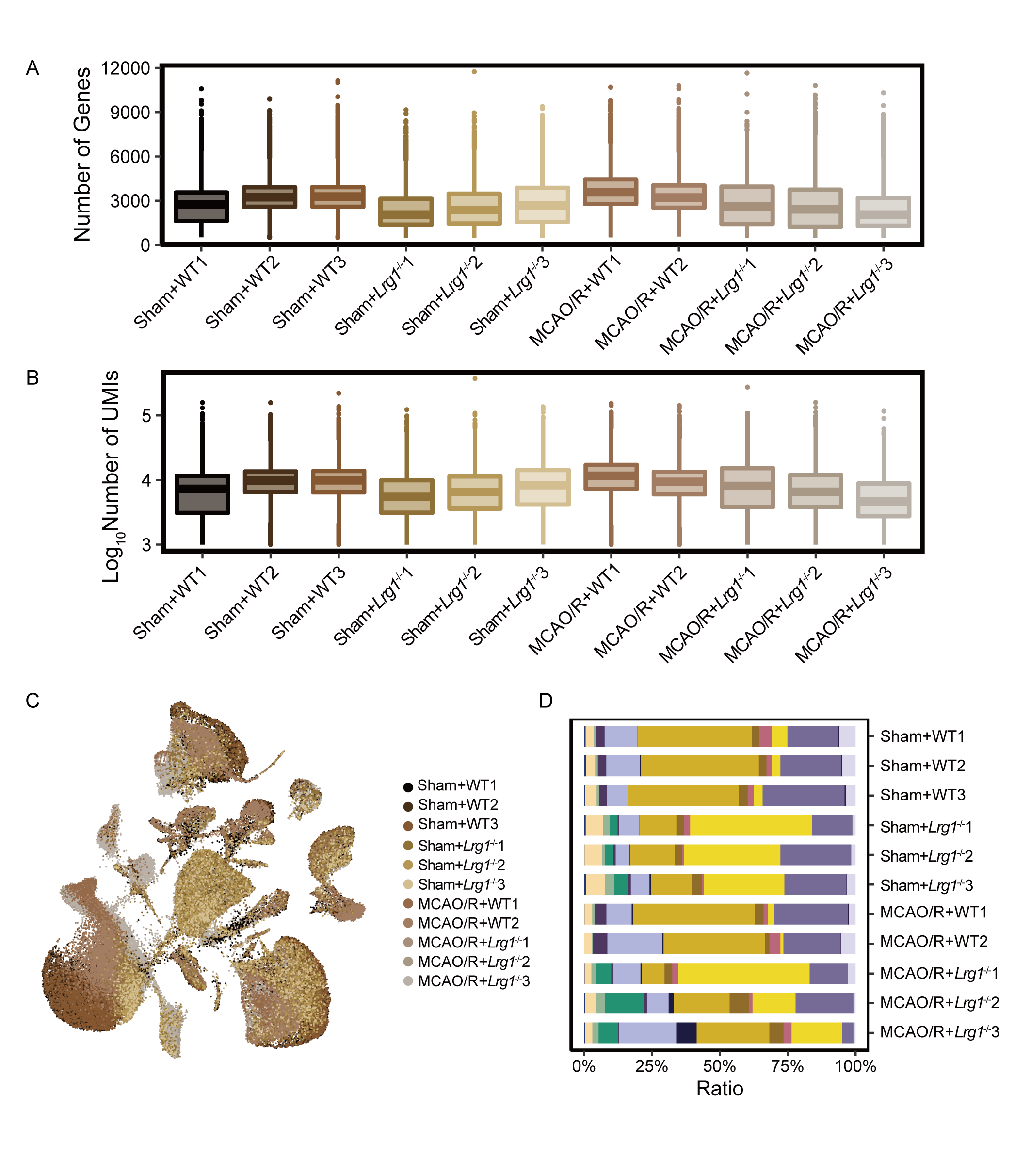

Supplement: Supplementary file 2 — Additional file 2: Figure S2. Basic information of single‒cell RNA‒seq data. [file 12974_2023_2941_MOESM2_ESM.png]

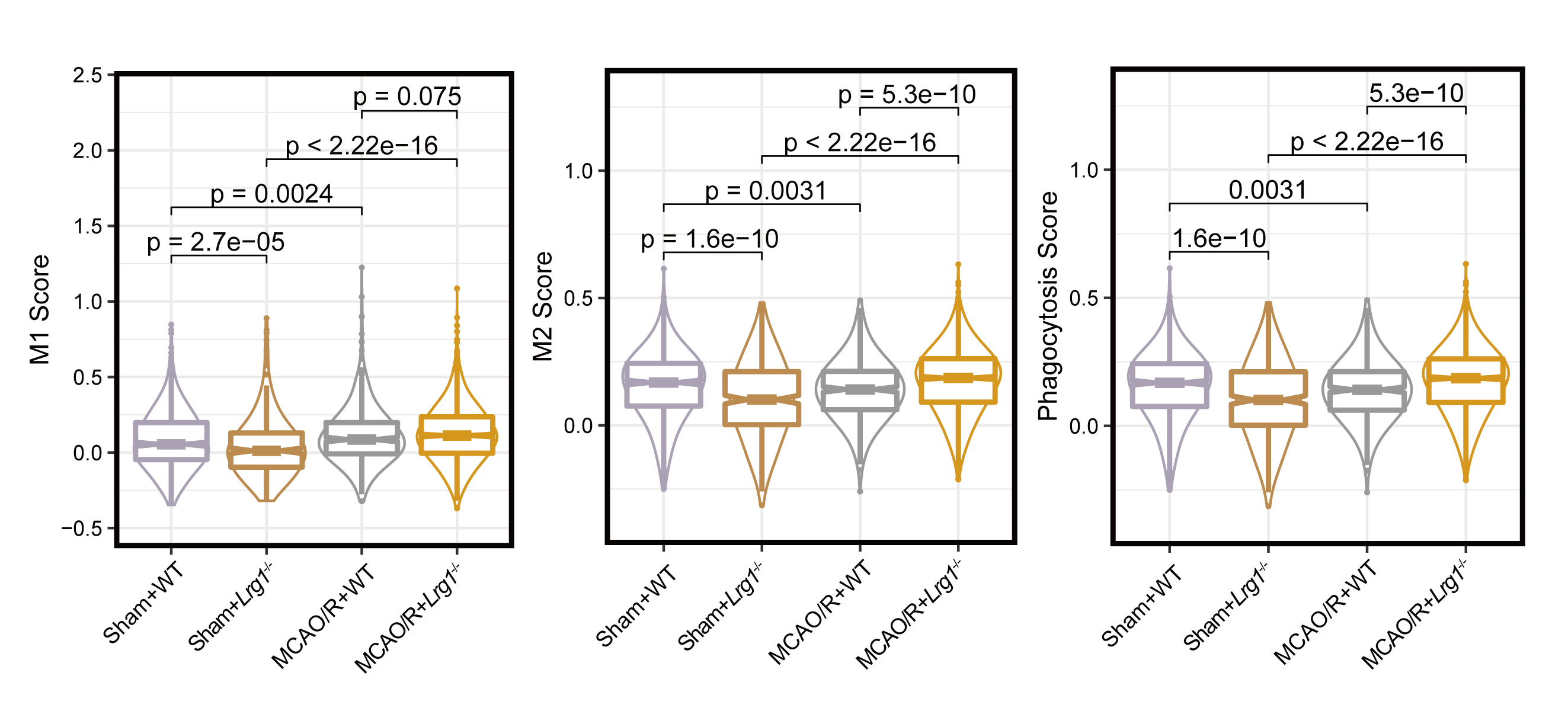

Supplement: Supplementary file 3 — Additional file 3: Figure S3. Functional status scores of macrophages in different groups. [file 12974_2023_2941_MOESM3_ESM.png]

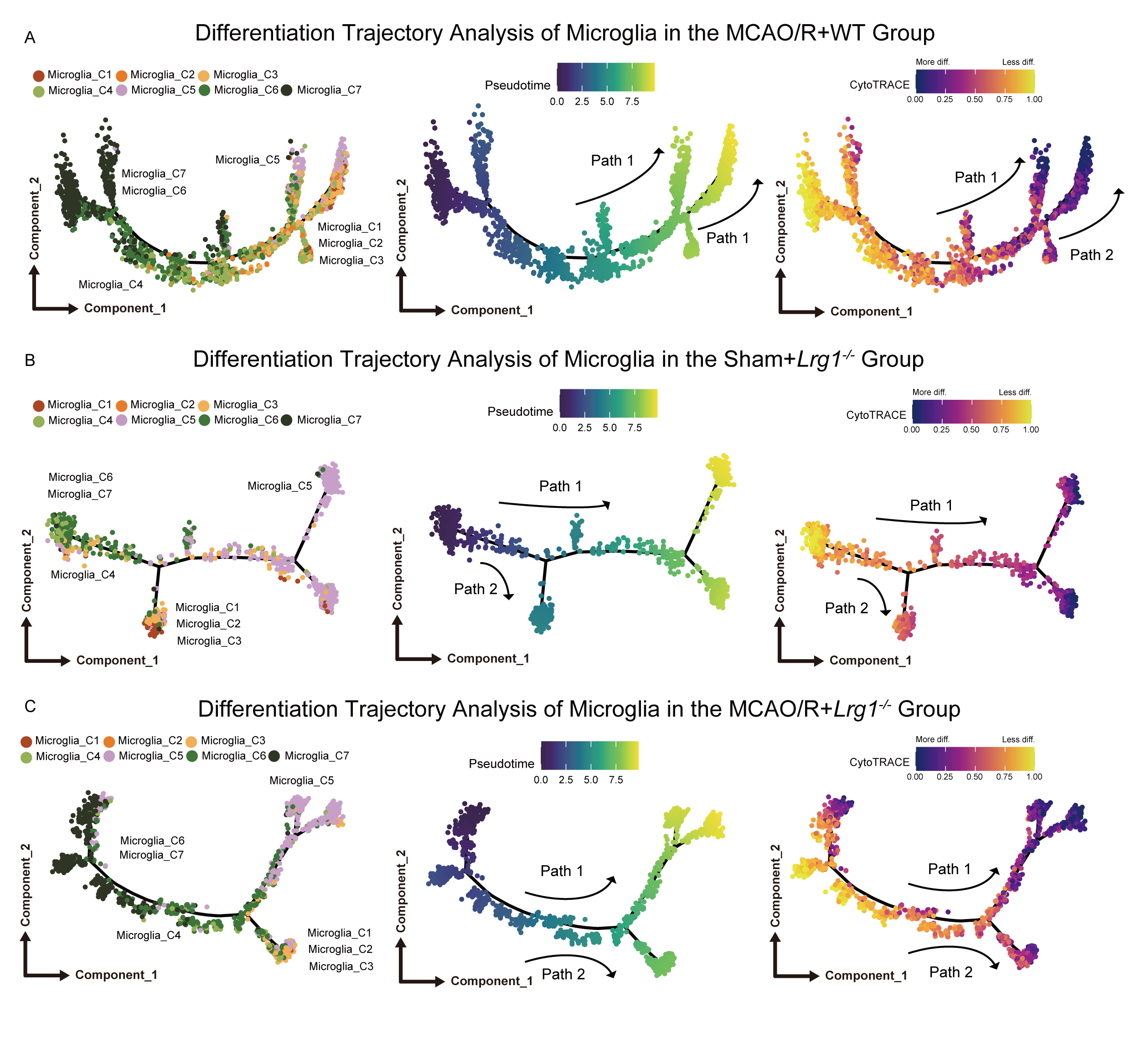

Supplement: Supplementary file 4 — Additional file 4: Figure S4. Differentiation trajectory analysis of microglia in the different groups. A Differentiation trajectory analysis of microglia in the MCAO/R + WT Group. Scatter plots depict distinct clusters (left), Pseudotime scores computed by Monocle 2 (center), and CytoTREACE scores (right), respectively. B Differentiation trajectory analysis of microglia in the Sham + Lrg1−/− Group. Scatter plots depict distinct clusters (left), Pseudotime scores computed by Monocle 2 (center), and CytoTREACE scores (right), respectively. C Differentiation trajectory analysis of microglia in the MCAO/R + Lrg1−/− Group. Scatter plots depict distinct clusters (left), Pseudotime scores computed by Monocle 2 (center), and CytoTREACE scores (right), respectively. [file 12974_2023_2941_MOESM4_ESM.png]

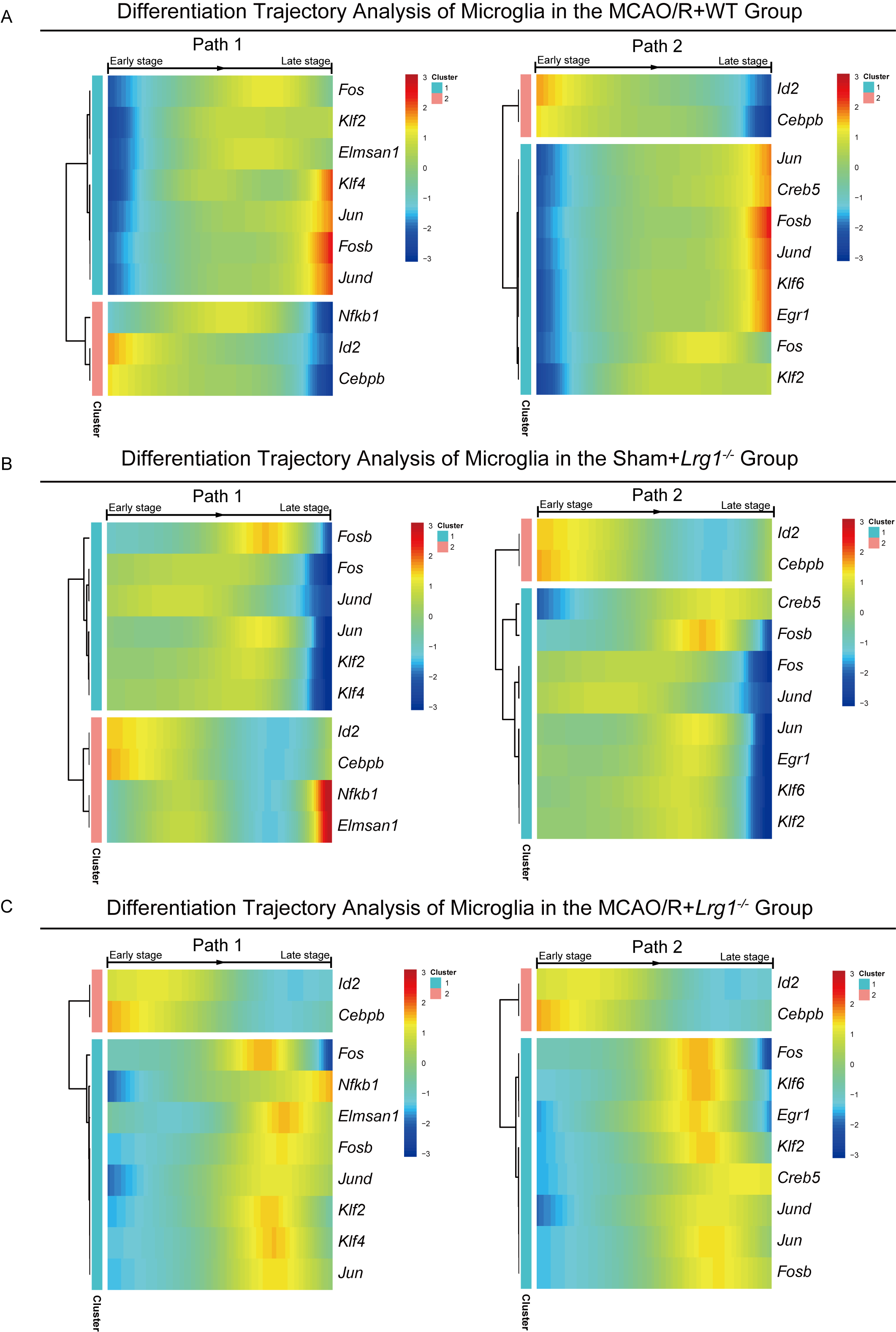

Supplement: Supplementary file 5 — Additional file 5: Figure S5. The transcription factors associated with microglial cell differentiation. A The expression profiles of the selected transcription factors, varying with pseudotime, were validated along two trajectories in the MCAO/R + WT Group. B Validation of the selected transcription factors' expression profiles, changing with pseudotime, along two trajectories in the Sham + Lrg1−/− Group. C Examination of the expression patterns of the selected transcription factors, modulated by pseudotime, along two trajectories in the MCAO/R + Lrg1−/− Group. [file 12974_2023_2941_MOESM5_ESM.png]

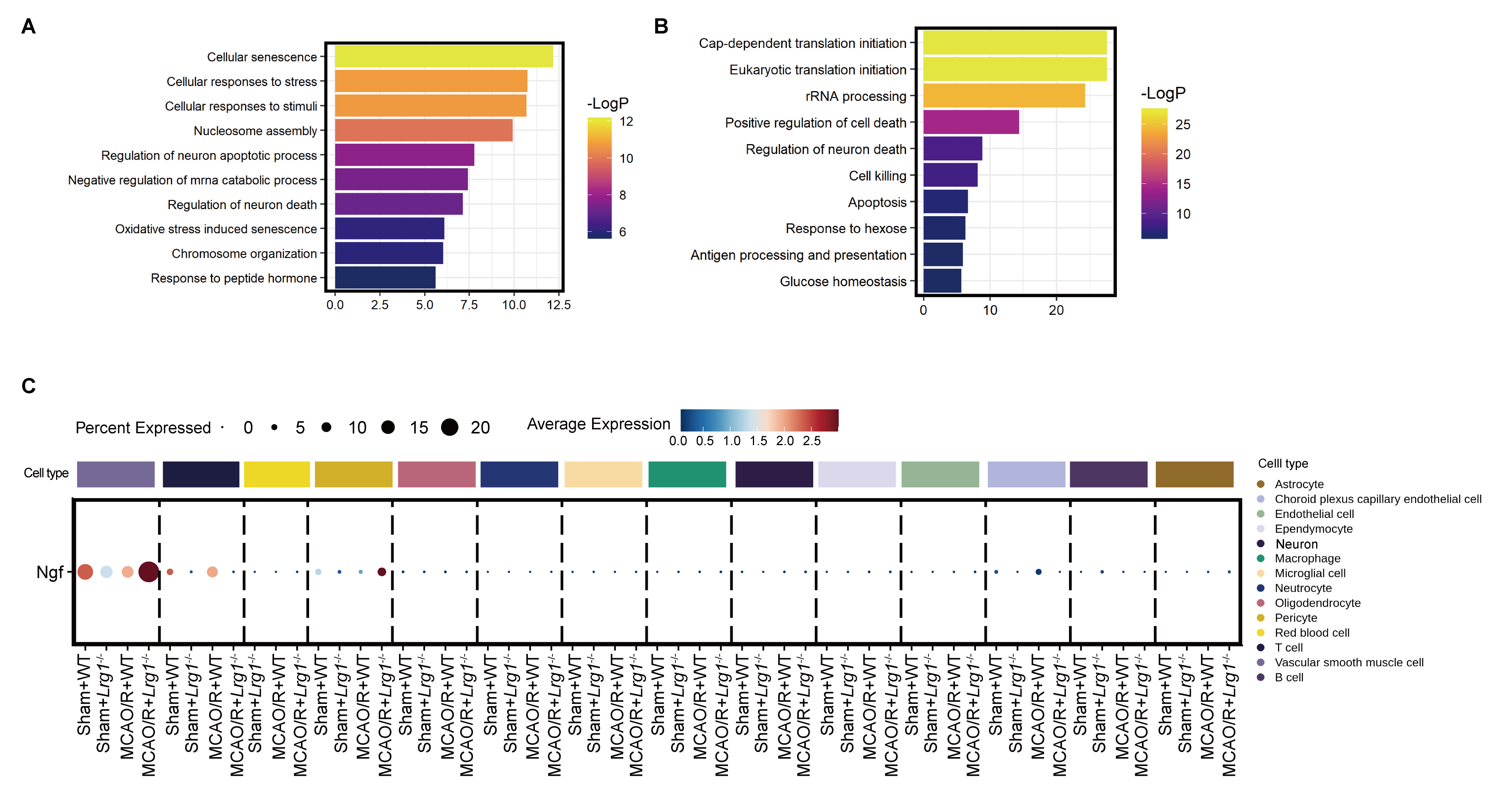

Supplement: Supplementary file 6 — Additional file 6: Figure S6. Functional enrichment analysis of differentially expressed genes upregulated in neurons and astrocytes of WT mice compared to Lrg1−/− mice. A. Functional enrichment analysis results of differentially expressed genes upregulated in neurons of WT mice compared to Lrg1−/− mice after cerebral ischemia‒reperfusion injury. B. Functional enrichment analysis results of differentially expressed genes upregulated in oligodendrocytes of WT mice compared to Lrg1−/− mice after cerebral ischemia‒reperfusion injury. C. Dot plot displaying the expression of Ngf in various cell types of brain tissues from different groups. [file 12974_2023_2941_MOESM6_ESM.png]
